# Ligand Independent and Subtype-Selective Actions of Thyroid Hormone Receptors in Human Adipose Derived Stem Cells

**DOI:** 10.1371/journal.pone.0164407

**Published:** 2016-10-12

**Authors:** Aleksandra Cvoro, Aleksandar Bajic, Aijun Zhang, Marisa Simon, Igor Golic, Douglas H. Sieglaff, Mirjana Maletic-Savatic, Aleksandra Korac, Paul Webb

**Affiliations:** 1 Genomic Medicine, Houston Methodist Research Institute, Houston, TX, United States of America; 2 Department of Pediatrics, Baylor College of Medicine, Houston, TX, United States of America; 3 Jan and Dan Duncan Neurological Research Institute at Texas Children's Hospital, Houston, TX, United States of America; 4 University of Belgrade - Faculty of Biology, 11000, Belgrade, Serbia; University Claude Bernard Lyon 1, FRANCE

## Abstract

Thyroid hormone (TH) receptors (TRs α and β) are homologous ligand-dependent transcription factors (TFs). While the TRs display distinct actions in development, metabolic regulation and other processes, comparisons of TRα and TRβ dependent gene regulation mostly reveal similar mechanisms of action and few TR subtype specific genes. Here, we show that TRα predominates in multipotent human adipose derived stem cells (hADSC) whereas TRβ is expressed at lower levels and is upregulated during hADSC differentiation. The TRs display several unusual properties in parental hADSC. First, TRs display predominantly cytoplasmic intracellular distribution and major TRα variants TRα1 and TRα2 colocalize with mitochondria. Second, knockdown experiments reveal that endogenous TRs influence hADSC cell morphology and expression of hundreds of genes in the absence of hormone, but do not respond to exogenous TH. Third, TRα and TRβ affect hADSC in completely distinct ways; TRα regulates cell cycle associated processes while TRβ may repress aspects of differentiation. TRα splice variant specific knockdown reveals that TRα1 and TRα2 both contribute to TRα-dependent gene expression in a gene specific manner. We propose that TRs work in a non-canonical and hormone independent manner in hADSC and that prominent subtype-specific activities emerge in the context of these unusual actions.

## Introduction

It is not clear why evolutionary processes selected for two distinct thyroid hormone (TH) receptor encoding genes. Major products of the THRA and THRB genes, TRα1 and TRβ1, are highly homologous ligand-dependent nuclear transcription factors (TFs) that partner with retinoid X receptors (RXRs) to regulate TH-dependent developmental decisions, metabolic homeostasis and other processes [1,2]. In both cases, unliganded receptor occupies specific TH response elements (TREs) and the active form of TH (triiodothyronine, T3) triggers exchange of receptor-associated coregulators and alters expression of TR target genes [3–5]. Analysis of TR knockout mice and humans and mouse models with TR mutations that cause TH resistance (RTH) reveals that TRs α and β regulate distinct physiological processes [6–8]. For example, TRβ1 acts in liver to regulate hepatic cholesterol and bile acid metabolism whereas TRα1 plays unique roles in regulation of heart rate, muscle physiology and bone development [9,10] and the two TRs also play distinct roles in cancer [11–13]. These TR subtype-specific effects, however, often correlate with TRα/TRβ ratios in individual cell types [9]. Further, dissection of TRα1 and TRβ1 action in cultured cells has only revealed moderate differences in transactivation and transrepression, homodimerization and DNA binding properties [14–16] and TRα1 and TRβ1 mostly regulate the same genes in native liver [17] and homologous cell types that express exogenous TRs [18–20]. Thus, differential effects of TRα1 and TRβ1 can be attributed to tissue/developmental stage-specific variations in TR expression but possible contributions of fundamental differences in TR subtype specific gene-regulatory properties are unclear [9,21].

Unliganded TRs are physiologically important [22,23] and display subtype-specific actions *in vivo* [24]. TRs are expressed before the onset of TH synthesis in frogs and mammals, implying hormone independent function [23,25,26], and unopposed actions of unliganded TRs in hypothyroidism and RTH results in deleterious effects on development, cancer incidence and metabolic response that are distinct from TR knockout [27]. Interestingly, TRα1, but not TRβ1, prevents precocious Xenopus metamorphosis [28]. Further, mice with defective PAX8, which regulates thyroid follicular cell genesis, exhibit congenital hypothyroidism and severe developmental defects which are selectively rescued by TRα1 knockout [27,29,30]. Again, the extent to which unliganded TR subtype selective effects are explained by differential TR expression or differences in TR mechanism is unclear.

THRA and THRB genes produce distinct spectrums of splice variant transcripts that encode TRs with unique functions [31,32]. TRβ2, a T3-binding TRβ splice variant that is expressed in a small subset of tissues, including pituitary and hypothalamus, is involved in regulation of the hypothalamic-pituitary-thyroid axis. The THRA gene encodes a major non-hormone binding TRα splice variant with a unique C-terminus (TRα2). TRα2 heterodimerizes with hormone binding forms of both TRs and exerts weak antagonistic effects on TH responses [31] and acts as phosphorylation-dependent single stranded RNA binding protein [33]. Currently, however, physiological significance of TRα2 is not clear.

THs and TRs can also act via non-genomic pathways, which are independent of intranuclear formation of T3-liganded or unliganded TR/chromatin complexes (reviewed in [34]). Some non-genomic TH-dependent effects are mediated by alternative TH-binding proteins, notably integrin αvβ3. However, TRα and certain transcriptionally inactive TRα splice variants, TRβ1 and TRβ1 RTH mutants have variously been implicated in regulation of mitochondrial activity, activation or modulation of second messenger cascades in different cell types and maintenance of actin cytoskeleton. Accordingly, TRs adopts a variety of extranuclear locations, including the mitochondrion, the inner surface of the cell membrane and throughout the cytoplasmic compartment.

While there is little evidence for large scale differences in TR subtype gene regulatory effects, there are reasons to suspect that TRs will prove to display different mechanisms of action *in vivo* [35]. Even though TRα1 and TRβ1 regulate similar gene sets in native liver and cultured cell types, there are TR subtype/gene-specific variations in responses to T3 and to unliganded TRs in these cells [3,18–20,36] and TRs even act in completely hormone-independent fashion at small subsets of genes in HepG2 and HeLa cells [18,19]. Moreover, ChiPseq studies reveal that TRα1 and TRβ1 sometimes occupy distinct chromatin regions [20]; while it has not yet been possible to link these TR binding events directly to subtype-specific genes [20], this finding suggests that TRs could influence distinct genes from distinct sites. Further, TRβ2 plays a central role in negative regulation of TH stimulating hormone (TSH) in cultured pituitary cells, even though TRα1 is present in the same cells and can subsume TRβ2 function after TRβ2 knockdown (KD) [37]. Finally, TR subtype specificity can emerge within the context of non-canonical TR actions [38,39].

Human adipose-derived stem cells (hADSC) are slow dividing multipotent adult stem cells that differentiate into a variety of TH-responsive cell types, including adipocytes, chondrocytes and osteocytes [40–43]. ADSC display low immunogenicity and no tumorigenicity and, unlike embryonic stem cells (ESC), there are few ethical concerns about use in humans. Thus, hADSC are potentially useful in cell-based therapies, tissue engineering and disease modeling. In this study, we set out to define TFs expressed in ADSC that may be important for multipotent phenotype. TRα predominates in hADSC, but not hADSC-derived differentiated cells, similar to our findings that TRα predominates in human ESC and induced pluripotent stem cells (iPSC) whereas TRβ transcripts are upregulated in mature iPSC-derived hepatocytes [44]. We find that both TRs are predominantly cytoplasmic and highly active in the absence of exogenous hormone in hADSC and that they influence cell division and hundreds of genes in a strongly TR subtype specific fashion. We suggest that prominent differences between TR subtypes can emerge in the context of unusual non-genomic actions and that unliganded TRs may function in similar ways in adult stem cells *in vivo*.

## Materials and Methods

### Reagents

Triiodothyronine (T3) was obtained from Sigma-Aldrich (Milwaukee, WI).

### Cell Culture

Human Adipose-Derived Stem Cells (ADSC) were purchased from Invitrogen (Invitrogen, Grand Island, NY, Cat. No. R7788115) and ZenBio (ZenBio Inc, Research Triangle Park, NC, Cat. No. ASC-F). All donors were non-diabetic females. Cells were maintained in Complete MesenPRO RS^™^ Medium (Invitrogen). Chondrogenesis was induced using STEMPRO^®^ Chondrogenesis Differentiation Kit according to manufacturer’s protocol (Invitrogen). After 21 days, cells were fixed, stained with Alcian Blue for proteoglycan content and phase contrast images were taken using an Olympus Ix81 microscope. Osteogenesis was induced using the STEMPRO^®^ Osteogenesis Differentiation Kit (Invitrogen) for 21 days and assessed using Alizarin Red staining to visualize calcium depositions in extracellular matrix. Adipogenesis was induced with STEMPRO^®^ Adipogenesis Differentiation Kit (Invitrogen) for 14 days and assessed using Oil-Red-O staining. Images were acquired at 4x and 10x magnification (Olympus Ix81 microscope).

### RNA Isolation

Following treatments indicated in figure legends, total RNA was prepared using Aurum Total RNA kit (Bio-Rad, Hercules, CA). Reverse transcription reactions were performed using 1 μg of total RNA with an iScript cDNA Synthesis kit (Bio-Rad).

### Microarray Analysis

Human HT-12_v4 whole genome expression arrays were obtained from Illumina (Illumina, San Diego, CA). cRNA synthesis and labeling were performed as described [44]. Results are deposited in the Gene Expression Omnibus (GEO) with accession numbers GSE75692 and GSE 75433. Arrays were scanned using the BeadArray Reader (Illumina). Unmodified microarray data obtained from GenomeStudio was background-subtracted and quantile-normalized using the *lumi* package [45] and analyzed with the *limma* package [46] within R software [47]. T3-response was determined by comparing cells treated with T3 (100nM) for 24 hrs against their respective untreated controls, and differentiation related changes by comparing differentiated cells with hADSC samples. The effect of TRα and TRβ KD was determined by comparing the siRNA control to both KDs respectively. Analysis was corrected for multiple hypothesis testing [48], and effects were considered significant when ≥2-fold with an adjusted p-value ≤ 0.05. To facilitate comparisons among various datasets, all data was uploaded into a SQLite3 database (http://www.sqlite.org/). Transcription Factors and associated partners were identified among the significantly affected genes through comparison to AnimalTFDB 2.0 [49].

### RT-qPCR

Real-time qPCR was performed with the Roche LightCycler 480 RT PCR Instrument using SYBR Green Mastermix (Roche, Mannheim, Germany). Sequences of the primers are available upon request. Data were collected and analyzed using the comparative threshold cycle method with GUSB, B2M, β-actin and 18S rRNA as reference genes. Experiments were performed at least three times, mean ± SD was calculated and statistical analysis was performed using the Prism curve-fitting program (GraphPad Prism, version 6.01). Expression of nuclear receptors was assessed using The Human Nuclear Receptors & Coregulators RT^2^ Profiler^™^ PCR Array (Qiagen, Hilden, Germany). Relative gene expression values were analyzed using the Superarray web-based software package performing all ΔΔ*C*_t_-based fold-change calculations.

### Transient Transfection

Cells were seeded onto 48-well plates in MesenPRO RS medium (Invitrogen) at 1.2x10^4^ cells per well and transfected the following day with luciferase reporters containing two copies of each TRE (DR4, F2) and FLAG-tagged TR expression vectors (Clontech Laboratories, Inc., Mountain View, CA) by using Xfect (Clontech Laboratories, Inc.) according to the manufacturer's instructions. 6h after transfection, media was changed (MesenPRO RS) and cells were treated with appropriate hormone dilutions (100nM T3 or GC-1). Twelve hours later, cells were lysed and assayed for luciferase activity (Promega, Madison, WI).

### Immunocytochemistry (ICC) and Fluorescence Imaging

ICC was performed on cells cultured in 4-well chamber slides (Nunc^™^ Lab-Tek^™^ II System, ThermoFisher Scientific, Carlsbad, CA). Cells were fixed with 4% PFA for 20 minutes, rinsed three times with PBS and then permeabilized in 0.1% triton dissolved in PBS for 10 minutes. Subsequently, cells were blocked for 30 minutes using 10% donkey serum in PBS.

For double immunostaining, we used TRα1 (1:100, PA1-211A, ThermoFischer Scientific), TRα2 (1:100, PA1-216, ThermoFischer Scientific), TRβ (1:250, sc-738, Santa Cruz, USA), COX IV, mitochondrial marker (1:100, ab14744, Abcam, UK) and calnexin, endoplasmic reticulum membrane marker (1:100, ab22595, Abcam, UK). All primary antibodies were diluted in PBS with 5% goat serum and applied directly into the chambers with cells. Antibodies used for double immunostaining were applied as a mixture with the exception of calnexin/TRα doublestaining which was sequential. Incubation was performed overnight at 4°C and then the antibodies were rinsed away three times in PBS with 1% bovine serum albumin. Cells were incubated with mixture of secondary antibodies Alexa Fluor 488 (1:400, A-11034, Life Technologies, Waltham, USA) and Alexa Fluor 633 (1:400, A-21052, Life Technologies) in PBS with 1% bovine serum albumin. After rinsing, the cells were labeled with nuclear stain Sytox Orange (1:1000, S11368; Life Technologies) for 5 minutes, washed two times in PBS and slides were then covered with Mowiol 488 and coverslip glass. Fluorescence imaging was performed on inverted Leica TCS SP5 II confocal microscope. Image analysis was performed in Leica LAS AF software. All images used for analysis were made on slide sets from two independent cell culture experiments with replicates.

Immunostaining for α-tubulin used mouse anti-α-Tubulin antibody conjugated with Alexa Fluor-488 (ThermoFisher Scientific) diluted 1:200 in PBS with 5% donkey serum and applied directly into the chambers with cells. Incubation was performed overnight at 4°C and then antibody was rinsed away three times in PBS with 1% bovine serum albumin. Additional labeling with DAPI (ThermoFisher Scientific) and Phalloidin-Alexa Fluor-647 conjugate (ThermoFisher Scientific—A22287, 5 U/ml) dissolved in 1% BSA in PBS took 20 minutes. Cells were rinsed at least 6 more times and slides were then covered with fluorescence mounting medium (DAKO, S3023) and coverslip glass. Fluorescence imaging was performed on inverted Zeiss LSM 710 confocal microscope with use of Zen 2010 software. Image analysis was performed in Zen 2010 and Imaris 8.1 software. All images used for analysis were made on slide sets from two independent cell culture experiments with replicates.

Cell size (cell surface area) and nuclear surface area were estimated from IF slides using Imaris 8.1 software. The non-parametric Kruskal-Wallis’s test followed by Dunn's post hoc test was used to identify significant differences. Statistical significance was accepted at *p*<0.05.

### RNA Interference

ADSC were plated in MesenPRO RS^™^ Medium and grown to 50% confluency. Cells were transfected with either TRα or TRβ ON-TARGET plus SMART pool siRNA, which contains an equal mix of different 4 siRNAs directed against the same transcript (Dharmacon, Waltham, MA), at 50 nM final concentration according to manufacturer’s protocol. Positive and negative non-targeting control siRNAs were also purchased from Dharmacon (Dharmacon). After two days, RNA was prepared for microarrays and qPCR. For 6-day experiments, cells were re-plated 48h and 96h after first siRNA transfection, and transfections were repeated in the same manner. To knock down particular TRα isoforms we used custom SMART pool ON-TARGET plus THRA1 or THRA2 siRNA (Dharmacon). Individual siRNAs were designated using the advanced Dharmacon tool (http://dharmacon.gelifesciences.com/design-center); 4 individual siRNAs with patented modifications to reduce off-targets were mix together to make a pool. All siRNA sequences are listed in S1 Table.

### Phase-contrast Microscopy

Cells were grown on 12-well culture dishes (BD Biosciences, Franklin Lakes, NJ), washed three times with PBS and examined under Nikon Ti-E microscope equipped with Cool SNAP HQ2 and DS-Fi1 cameras. Representative images were taken at 4X, 10X and 20X magnification.

### Western Blotting

Total hADSC protein was concentrated by lyophilization and separated with 4%–12% gradient Bis-Tris gels (Invitrogen). Proteins were transferred to PVDF membranes (Bio-Rad) and incubated with anti-TRα1 (ThermoFisher Scientific, Cat. No. PA1-211A) or anti-TRα2 (ThermoFisher Scientific, Cat. No. PA1-216) at a concentration of 1:1000. The Luminata Forte Western HRP Substrate (EMD Millipore, Billerica, MA) was used for protein detection.

### Function Enrichment Analysis (GeneCodis)

We used GeneCodis analysis (http://genecodis.dacya.ucm.es/) to integrate differentially expressed genes to find groups of genes with similar biological meaning and identify enriched functional themes. GeneCodis is a function analysis tool for singular and modular enrichment analysis which integrates different information resources (GO, Panther pathways, SwissProt, etc.) [50–52]. Genes of interest, defined as at least 2-fold differentially expressed according to microarray analysis, were uploaded into the application as standard human gene symbols and genes in the interaction network with FDR <0.05 were taken into consideration.

### Ingenuity Pathway Analysis (IPA)

For Ingenuity Pathway Analysis (Ingenuity Systems, Redwood City, CA), genes of interest, defined as those at least 2-fold differentially expressed, were uploaded and overlaid onto a global molecular network developed from the Ingenuity Pathways Knowledge Base (IPKB). The IPKB was searched to find sub-networks enriched in genes of interest. Significance for biological functions was assigned to each network by determining a *p*-value for the enrichment of the genes in the network for such functions compared with the whole IPKB as a reference set.

### GeneMANIA

We used GeneMANIA (http://www.genemania.org) to identify genes related to sets of input genes, identified as TRα or TRβ targets [53–55]. Genes used as input were differentially expressed genes underlying specific functional themes as identified by microarray, GeneCodis and IPA. The GeneMANIA algorithm comprises a linear-regression-based algorithm for calculating single, composite functional association networks from multiple networks derived from different proteomic or genomic data sources; and prediction of gene function. We focused the analysis only on high confidence physical interactions (from various protein interaction databases included in GeneMania) and pathway interactions (from Reactome pathway database). The resulting sub-network containing our query genes and additional related genes helps interpret mechanistic details of the functional themes we define.

### Flow Cytometry

Cells were collected and fixed in 70% ethanol for at least 2 hours at -20°C. DNA was stained with DAPI (1 μg/ml) and cells analyzed on LSR II instrument with DIVA 8.0 software. Final analysis was performed with FlowJo 10.1 software.

## Results

### Changes in TR Subtype Expression in hADSC Differentiation

**Fig 1:**
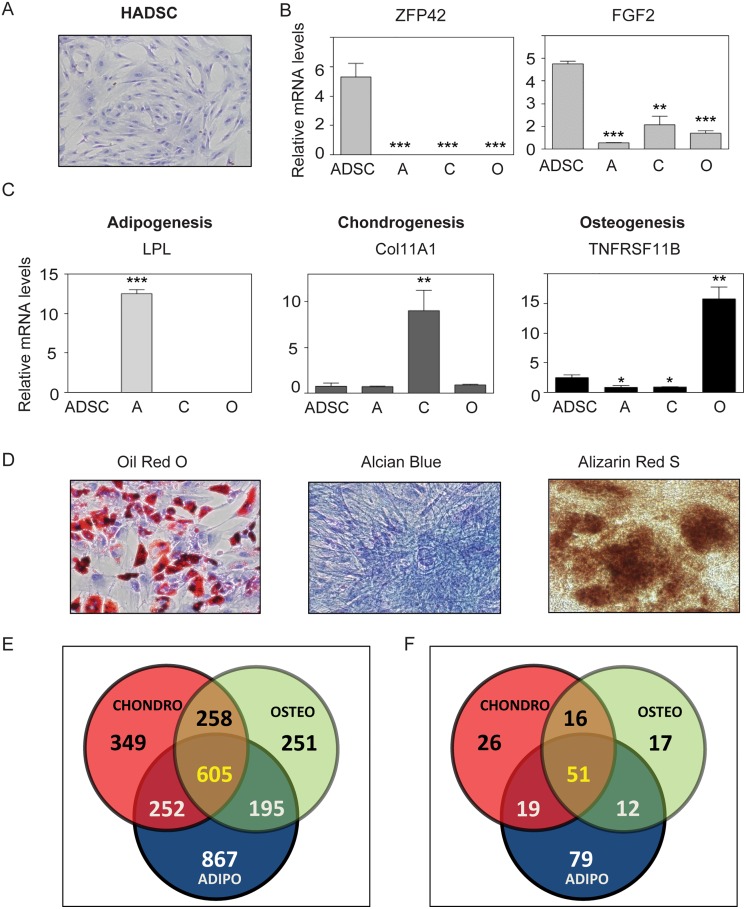
Verification of hADSC differentiation along adipogenic, chondrogenic or osteogenic paths. (A) Image of hADSC. (B, C) qRT-PCR analysis showing decreased expression of stem cell markers (B) and emergence of specific differentiation markers (C). The error bars represent the SD. Asterisks show statistically significant changes (***, *p* < 0.001; **, *p* < 0.01; *, *p* < 0.05). (D) Stained images of cells confirming appropriate differentiation. Venn diagrams representing differential gene expression after adipogenesis, chondrogenesis and osteogenesis as revealed by microarray (E) and transcription factor (TF) analysis.

**Fig 2:**
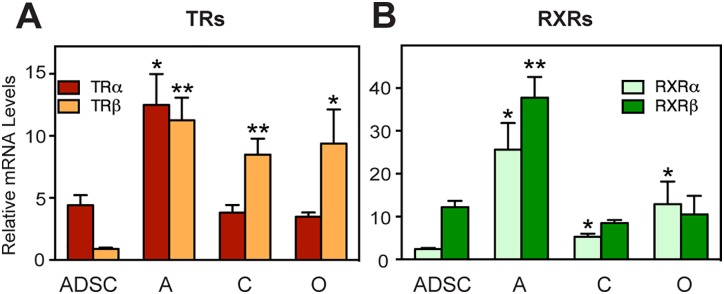
TR subtype switching in hADSC differentiation. (A, B) Expression of TRs and RXRs assessed by qPCR. Data represented as mean ± SD. Statistical significance of the observed changes is denoted by asterisk. (*p* < 0.01; *, *p* < 0.05). A = adipocytes; C = chondrocytes; O = osteoblasts.

We analyzed TF expression in human (h) ADSC before and after their differentiation along the adipogenic, chondrogenic and osteogenic lineages (Fig 1A–1D). Microarray analysis revealed 1919, 1464 and 1309 genes display differential expression in hADSC versus each differentiated cell type and that 605 displayed common changes in all three lineages (Fig 1E). The latter group encompassed 51 transcription-related factors, including nuclear receptor (NR) COUP-TF1 (Fig 1F, S2 Table). We therefore assessed expression of all NRs during hADSC differentiation using more sensitive commercial PCR arrays (S3 Table). This approach revealed that TRβ1 and RXRα were also upregulated in hADSC-derived lineages; whereas retinoic acid receptor (RAR) γ was down-regulated. Independent qPCR validation confirmed that TRα transcripts were modestly upregulated in adipogenesis but unchanged in chondrocytes and osteoblasts and that TRβ1 and RXRα transcripts were upregulated in all three differentiated lineages (Fig 2A and 2B).

Interestingly, hADSC expressed TRα1 and TRα2 transcripts (S1 Fig). Both TRα1 and TRα2 transcripts were coordinately upregulated during adipogenesis, similar to total TRα (Fig 2). However, TRα1 was selectively upregulated during differentiation to chondrocytes and osteocytes while there were no statistically significant changes in TRα2 expression (S1A and S1B Fig). The importance of these differential effects is unclear.

### T3 does not Influence TR Target Gene Expression in hADSC

To understand TR function in hADSC, we first assessed their capacity for T3 response. Unexpectedly, we failed to detect effects of T3 (100nM) on gene expression in parental hADSC using microarray analysis (data deposited in NCBI’s Gene Expression Omnibus; GSE75433). Similar results were obtained in two independent pools of cells from six donors, suggesting that lack of T3 response is unrelated to donor/batch variability. To partially address the possibility that microarray analysis was not sensitive enough to detect T3 regulated genes in this cell background, we performed targeted qPCR analysis of known TR target genes that commonly respond to T3 in multiple cell types including hairless (HR) and Kruppel-Like Factor (KLF) 9 ((18,44): S2A Fig). Neither gene responded to T3 in parental hADSC but did respond to 100nM T3 in hADSC-derived adipocytes. We also verified that hADSC are capable of mounting responses to T3 (100nM) and the thyromimetic GC-1 at a dose that is saturating for both TRs (100nM) after transfection of TRs and TRE-reporters (S2B Fig). Thus, there is no general defect in TR signaling or hormone import to explain lack of T3 response in this cell type and it seems likely that the inability to respond to T3 is a property of endogenously expressed receptors.

Further exploration of T3 response in hADSC-derived adipocytes, chondrocytes and osteoblasts using microarray analysis revealed multiple T3 regulated genes in addition to HR and KLF9 (S2C Fig; genes listed in S4, S5 and S6 Tables). In adipocytes, T3 induced 10 genes and repressed more than 40, in chondrocytes 18 genes were upregulated and 19 downregulated and 59 were upregulated and 78 down regulated in osteocytes. T3-regulated genes included documented verified or possible TR target genes, including TP53I11 [18,56], PPARGC1A [57] and APOE [58] in osteocytes and IGF1 [59] and DACT1 [44] in chondrocytes. There was little overlap between T3 targets in adipocytes, chondrocytes and osteocytes (S2C Fig) and the relatively small number of hormone-responsive genes means that we cannot readily predict effects of T3 on particular pathways and processes in each hADSC-derived lineage. The fact that T3 response emerges after hADSC differentiation nevertheless underscores the notion that there is no intrinsic genetic block to T3 action in parental hADSC. Further, the fact that increases in TRβ levels correlate with emergence of the capacity for T3 response raises the possibility that elevated TRβ levels could be one factor that permits TRs to act in canonical fashion in this cell type.

### hADSC TRs are Predominantly Cytoplasmic

To understand lack of T3 response in hADSC, we used immunofluorescent (IF) labeling to determine whether we could detect TR protein and assess subcellular localization (S3 Fig). Double-IF labeling of either TRα1 (green, upper panels) or TRα2 (green, lower panels) along with TRβ (red) revealed detectable staining for all three forms of TR. Specificity of labeling was ensured in control experiments that omitted primary antibodies (S4 Fig). Remarkably, all TRs adopted primarily cytoplasmic immunolocalization. TRα1 displayed strong cytoplasmic localization, with some nuclear staining. TRα2 displayed weaker overall staining than TRα1, but nevertheless displayed a similar predominantly cytoplasmic distribution. TRβ staining was very weak, in accordance with low transcript levels, but appeared exclusively cytoplasmic. We were unable to detect colocalization of either form of TRα with TRβ (right panels).

**Fig 3:**
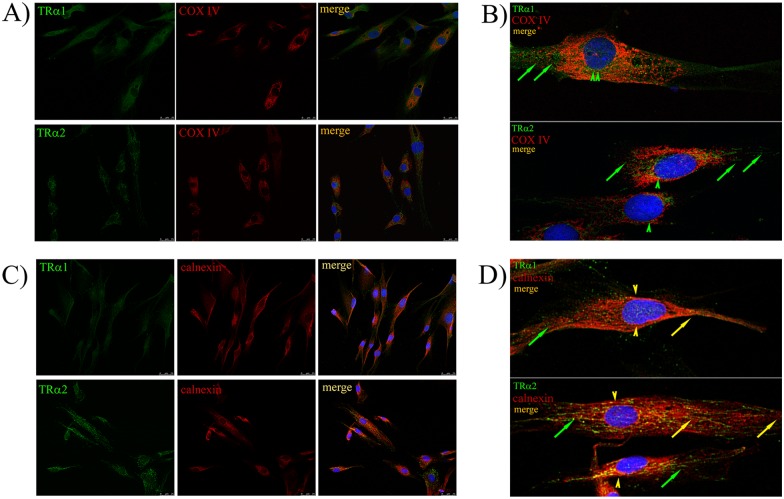
Subcellular partitioning of TRα1 and TRα2 in hADSC to mitochondria and endoplasmic reticulum. Confocal images of hADSC showing double immunostaining for TRα1 and TRα2 (green) with mitochondrial marker COX IV (red—A, B) or endoplasmic reticulum marker—calnexin (red—C, D). Magnified view of TRα1 and TRα2 localization in cisternal structures (endoplasmic reticulum) at the cell periphery (green arrows) and perinuclear region (green arrowheads) (B) and their colocalization with calnexin—(D—yellow arrows, yellow arrowheads). Green arrows on D) pointing to TRα1 and TRα2 localization outside of endoplasmic reticulum. Bar: A, B = 25 μm.

Further assessment revealed TRα immunolocalization in cytoplasmic membranous subcompartments (Fig 3). Double immunostaining of TRα1 and TRα2 (green) with the mitochondrial marker COX IV (red) was consistent with a predominantly mitochondrial localization for TRα proteins (Fig 3A), as noted in other cell types (see [60]). We also observed TRα immunolocalization in cisternal structures at the cell periphery (Fig 3B, green arrows) and perinuclear area (Fig 3B, green arrowheads). We confirmed that this corresponded to endoplasmic reticulum (ER) with double immune labeling of TRα1 and TRα2 (green) with the ER marker calnexin (red) (Fig 3C and 3D). A magnified view of TRα1 and TRα2 colocalization with calnexin at the cell periphery and perinuclear region (Fig 3D, yellow arrows and yellow arrowheads respectively) also indicated that TRα1 and TRα2 were localized outside of endoplasmic reticulum (Fig 3D, green arrows).

### hADSC TRs are Active without Hormone and Exhibit Subtype-Specific Effects on Cell Morphology

We used siRNA to learn whether hADSC TRs were active in the absence of exogenous hormone. Initial experiments utilized a verified commercially available pool of four highly specific siRNAs for each TR transcript, which allows us to use low concentrations of reagent and reduces the possibility of cross-reaction with unrelated mRNA species (Materials and Methods). Additionally, the siRNAs that we used are dual strand modified to favor antisense strand uptake and to destabilize off target activity and enhance target specificity [61]. Specific KD of total TRα was verified by qPCR (S5A Fig) and western analysis confirmed that levels of TRα1 and TRα2 immunoreactive species were diminished after TRα siRNA treatment (S5B and S5C Fig). Further, TRα1 IF was abolished after siRNA transfection and TRα2 immunofluorescence was markedly diminished (S6 and S7 Figs), parallel to effects of siRNA treatment in westerns. Likewise, TRβ transcripts were reduced after TRβ siRNA treatment (S5A Fig). Although we did detect TRβ protein by IF, we were unable to detect immunoreactive TRβ1 protein in hADSC by westerns, probably because TRβ1 antibodies are insufficiently sensitive to detect low levels of TRβ by western [62]. However, we did observe complete abolition of TRβ IF signal after TRβ siRNA treatment (S6 and S7 Figs).

**Fig 4:**
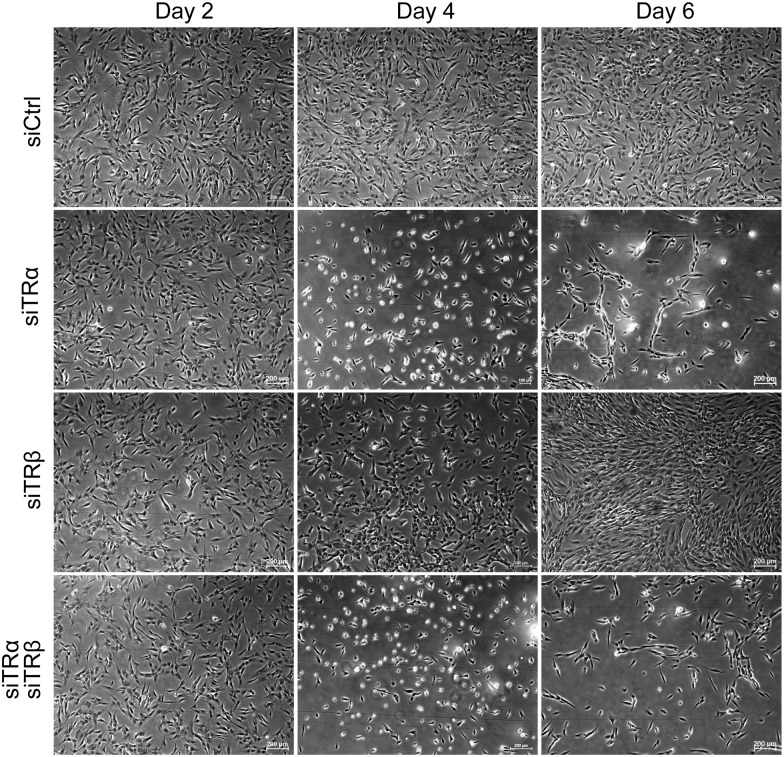
Effects of TR silencing on hADSC. hADSC were repeatedly treated with siTRα or/and siTRβ over 6 days. Cells were examined with Nikon Ti-E microscope (magnification 4x).

**Fig 5:**
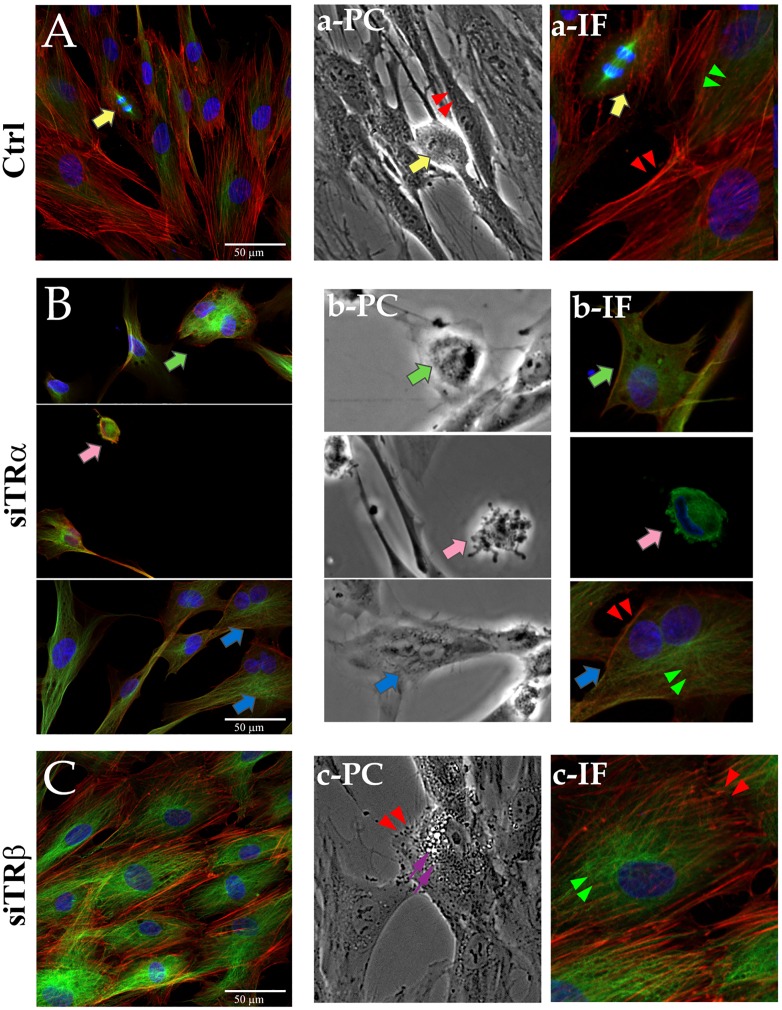
TR silencing alters hADSC morphology. Confocal images of hADSC (A, a-IF), siTRα (B, b-IF) and siTRβ (C, c-IF) showing immunostaining for actin (red), α-tubulin (green) and DAPI DNA counterstaining. Panels on right represent details from the left images examined at confocal (IF) and phase-contrast (PC) microscopes. Mitosis-yellow arrows; mitotic cell rounding/mitotic arrest (green arrows); apoptotic cell (pink arrows); binuclear cell (blue arrow); lipid droplets (violet arrow); actin (red arrowheads); tubulin alpha (green arrowheads). Bar: A, B, C = 50 μm.

Remarkably, TR KD resulted in striking changes in cell morphology, which were highly TR subtype specific (Fig 4). While control cells retained their characteristic long, thin spindle-shaped fibroblastic morphology, TRα KD resulted in rounding and detachment of cells (Figs 4 and 5). By contrast, TRβ KD resulted in the appearance of rhomboidal and stellate cells. Joint TRα/TRβ KD revealed that the TRα KD phenotype is dominant. We have performed KDs of many other TFs in hADSC (manuscript in preparation), including other NRs that displayed differential regulation during hADSC differentiation pathways, Ets translocation variant (ETV) 5, multiple Kruppel-like Factors (KLFs), the corepressor SIN3A and others. We have not observed comparable morphological changes in any of these cases.

We further analyzed appearance of TR KD cells using confocal microscopy with IF labelling of actin (red), tubulin-α (green) and DAPI DNA counterstaining (Fig 5, S8A Fig) and also counted cells in different phases of the cell cycle and determined total nuclear and cell surface area (Table 1, S8B and S8C Fig). Whereas control hADSC displayed normal mitosis (Fig 5A, S8A Fig, yellow arrows), TRα KD cells variously displayed signs of arrested mitosis, including rounding with lack of obvious chromosomes and mitotic spindle (Fig 5B, green arrow), and apoptosis (Fig 5B, pink arrow). We also detected (>16%) binuclear cells (Fig 5B, S8A Fig, blue arrows; Table 1) and a reduction in total cell and nuclear size (S8B and S8C Fig). In contrast, TRβ KD cells displayed similar mitotic index (MI) to control hADSC and showed increases in cell and nuclear size (Fig 5, S8 Fig; Table 1). TR KD also led to a more organized cytoskeletal network with dense actin fibers at cell edges (Fig 5C, red arrowheads), distinct from control hADSC which maintain typical mesenchymal actin fiber organization throughout the cell (Fig 5A, red arrowheads). We also observed changes in cytoskeletal content; TR KD promoted formation of a tubulin based cytoskeleton (Fig 5, green arrowheads). Finally, we found examples of TRβ KD cells with prominent lipid droplet accumulation, a hallmark of adipogenesis (Fig 5C, purple arrowheads).

**Table 1:** Effects of TR silencing on cell division. Mitotic index, the percentage of mitosis stage and binuclear cells were counted in Zen 2010.

	Cells in interphase (%)	Mitotic index (%)	Mitotic cells in prometaphase/metaphase (%)	Mitotic cells in anaphase/telophase (%)	Binuclear cells (%)
**siCtrl**	93.01	6.9	42.1	57.9	0
**siTRα**	81.53	0	0	0	16.79
**siTRβ**	91.3	8.2	46.7	53.3	0.54

### TRα and TRβ Regulate Distinct hADSC Gene Sets

**Fig 6:**
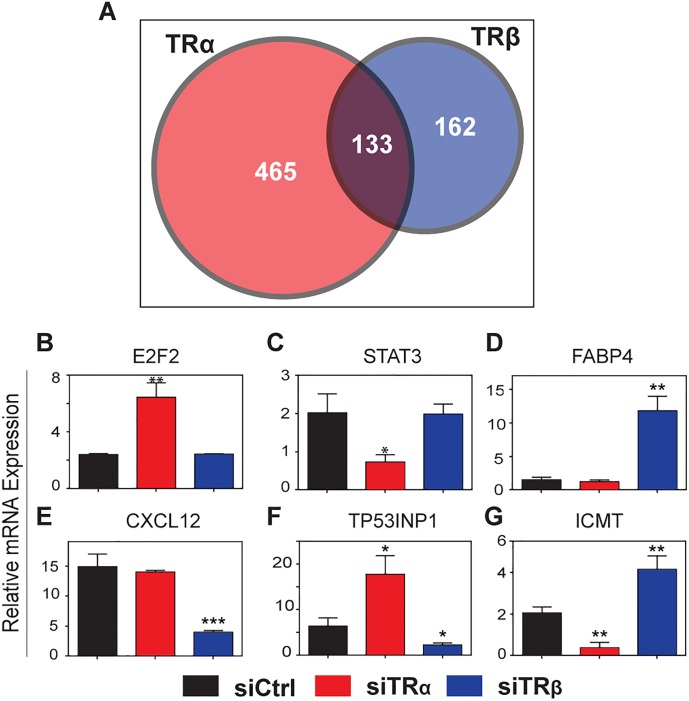
Unliganded TRα and TRβ regulate distinct genes in hADSC. (A) Differential gene regulation in hADSC cells after TRα and TRβ KD. (B-G) Effects of TRα and TRβ KD at representative target genes. All data are represented as mean ± SD. ***, *p* < 0.001; **, *p* < 0.01; *, *p* < 0.05.

To understand gene expression changes associated with TR KD-dependent changes in cell appearance, we performed microarray analysis two days after TR siRNA treatment of hADSC, before prominent morphological changes fully emerge (see Fig 4). We detected large numbers of TR regulated genes (Fig 6A). TRα KD resulted in ≥2-fold expression change of 598 genes, with 345 upregulated (58%) and 253 downregulated (42%). TRβ KD resulted in 295 changes, with >70% of genes upregulated. Remarkably, only 133 genes were commonly regulated; a level of overlap that is similar to TRs and completely unrelated TFs, such as ETV5 (S9 Fig).

Some putative hADSC TR target genes respond similarly to TRs in other cell lines. For example, unliganded TRα also suppressed JARID2 and E2F2 expression and both TRs suppressed FOSL1 expression in HepG2 [18,20,56]. Despite the inability of these genes to respond to T3 in hADSC, they were T3-inducible in HepG2 and another cell line, C17.2 cerebellum cells [18–20]. Additional documented TR and T3 target genes that responded to TR KD in hADSC included ANGPTL4, SLC16A6, ITGA2, CITED4, BATF3, FNDC5 [18,62].

We confirmed specificity of response to TR KD by qRT-PCR (Fig 6, S10 Fig). Genes that were selectively de-repressed by TRα KD included TFs involved in cell cycle regulation such as E2F2 and the aforementioned JARID2, cyclin E2 (CCNE2), which plays a role in G1/S transition and HIST1H2BD and other histones (Fig 6B, S10A–S10D Fig). Genes repressed by TRα KD (Fig 6C, S10E–S10G Fig) included the TF STAT3, the erk kinase MAPK3 and myosin light chain kinase MYLK. TRβ KD (Fig 6D and 6E; S10H–S10J Fig) also led to specific induction of multiple genes, including lipid storage genes (FABP4, CIDEA and SOAT2) and osteopontin (SPP1), and repression of the chemotactic factor CXCL12 (Fig 6E). Some genes that were commonly regulated by TRα and TRβ KD displayed similar responses to TRs (GLI1, S10K Fig), but many were oppositely regulated, including antiproliferative and proapoptotic p53 inducible protein TP53INP1, isoprenylcysteine carboxyl methyltransferase (ICMT) and matrix metallopeptidase 9 (MMP9) (Fig 6F and 6G; S10L Fig). Thus, TRα and TRβ regulate distinct gene sets in the absence of hormone in this cell background.

We verified that some observed TRα siRNA pool-specific effects on gene expression were consequences of TR KD. We obtained three individual siRNA from the TRα-specific smartpool and determined that each one reduced TRα transcript levels. Examination of effects of these siRNA on selected TRα specific genes confirmed similar effects in all three cases at the NEK2 and ICMT genes and in two of three cases with a strong trend towards significance in the other case at the TP53INP1 and STAT3 genes (S11 Fig). Thus, it is unlikely that these TRα-dependent effects are related to off-target interactions of siRNA.

### TRα Regulates hADSC Cell Cycle

Investigation of TR-regulated gene function revealed that TRs influence distinct hADSC processes and pathways. GeneCodis analysis revealed that both TRs were involved in cell-cell signaling, signal transduction and other processes (Table 2, S7 Table) but mostly regulate different genes within these categories (S7 Table). TRα also influences mitosis, cell division, chromosome segregation and other cell cycle associated processes (Table 2, highlighted). TRβ KD did not affect specific TRα-independent processes.

**Table 2:** TR mediated processes. Canonical processes obtained from GeneCodis using SlimProcess database. Gene co-occurence annotation found by Genecodis for the genes differentially expressed (FC > 2, *P* < 0.05 corrected for multiple testing) between siCtrl versus siTR hADSC samples. *P*-values have been obtained through hypergeometric analysis (Hyp) corrected by FDR method (Hyp*). Microarray data have been deposited in NCBI’s Gene Expression Omnibus; accession number GSE75692.

NGR	NG	Hyp	Hyp*	Annotations	
**TRα mediated processes**					
242	21	1.33E-10	4.92E-09	GO:0007267	cell-cell signaling (BP)
1176	43	8.31E-08	1.54E-06	GO:0007165	signal transduction (BP)
187	13	5.37E-06	6.62E-05	GO:0007067	mitosis (BP)
286	16	7.59E-06	7.02E-05	GO:0051301	cell division (BP)
312	16	2.22E-05	0.000164529	GO:0008283	cell proliferation (BP)
59	7	2.78E-05	0.000171288	GO:0007059	chromosome segregation (BP)
630	23	9.16E-05	0.000483971	GO:0055085	transmembrane transport (BP)
155	10	0.000120636	0.000495946	GO:0006950	response to stress (BP)
34	5	0.000141915	0.000525084	GO:0040007	growth (BP)
556	21	0.000114994	0.000531848	GO:0007155	cell adhesion (BP)
200	11	0.000229267	0.000771169	GO:0034641	cell nitrogen compound metabolic process (BP)
435	17	0.000341011	0.00105145	GO:0007049	cell cycle (BP)
73	6	0.000788391	0.00224388	GO:0030198	extracellular matrix organization (BP)
519	17	0.00232029	0.0061322	GO:0030154	cell differentiation (BP)
150	7	0.00753011	0.0185743	GO:0006464	protein modification process (BP)
128	6	0.0126971	0.029362	GO:0009790	embryo development (BP)
**TRβ mediated processes**					
242	12	4.09E-05	1.19E-05	GO:0007267	cell-cell signaling (BP)
556	16	6.87E-06	9.96E-05	GO:0007155	cell adhesion (BP)
1176	22	0.000112179	0.00108439	GO:0007165	signal transduction (BP)
312	10	0.000156698	0.00113606	GO:0008283	cell proliferation (BP)
519	13	0.000199073	0.00115462	GO:0030154	cell differentiation (BP)
128	6	0.000464644	0.00224578	GO:0009790	embryo development (BP)
630	11	0.00953599	0.0307271	GO:0055085	transmembrane transport (BP)

NGR = Number of annotated genes in the reference list; NG = Number of annotated genes in the input list; Hyp = Hypergeometric pValue; Hyp* = Corrected hypergeometric pValue

Ingenuity Pathway Analysis (IPA) confirmed that TRα target genes associate with cell cycle-related functional themes and also revealed involvement in all phases and phase checkpoints (Table 3). By contrast, TRβ was only involved in cell cycle progression, S phase and entry into S-phase. Even within these functional themes, TRβ regulated mostly different genes from TRα (S8 Table). The vast majority of these TRα responsive genes are de-repressed after KD (S8 Table red), implying that TRα plays an important role in suppressing genes involved in hADSC cell division.

**Table 3:** Functional categorization of TR target genes in hADSC. Pathway enrichment determination using Ingenuity pathway analysis identify enriched cell cycle-related functional themes. The number of genes and statistical values are shown for each TRα and TRβ knockdown in hADSC. Microarray data have been deposited in NCBI's Gene Expression Omnibus (GEO); accession number GSE75692.

		TRα		TRβ	
Function	Function annotation	NG	p-value	NG	p-value
Cell cycle progression	Cell cycle progression	68	0.0000001	35	0.000132
S phase	S phase	23	0.0000029	14	0.000045
S phase	Entry into S phase	14	0.0000388	10	0.000036
Cell cycle progression	re-entry into cell cycle progression of tumor cell lines	3	0.000318		
M phase	M phase	20	0.0000205		
M phase	arrest in M phase	8	0.0000372		
mitosis	mitosis	37	0.0000014		
mitosis	arrest in mitosis	9	0.000549		
interphase	arrest in interphase	32	0.0000017		
interphase	interphase	42	0.000017		
G1 phase	arrest in G1 phase	21	0.0000173		
G1 phase	G1 phase	26	0.000189		
cyokinesis	cytokinesis	15	0.000239		
prometaphase	arrest in prometaphase	5	0.0000266		
senescence	senescence of cells	19	0.0000511		
replication	replication of bone marrow cells	3	0.000162		
formation	formation of mitotic spindle	8	0.000342		
premature senescence	premature senescence of fibroblasts	4	0.00035		
endoreduplication	endoreduplication	4	0.000468		

**Fig 7:**
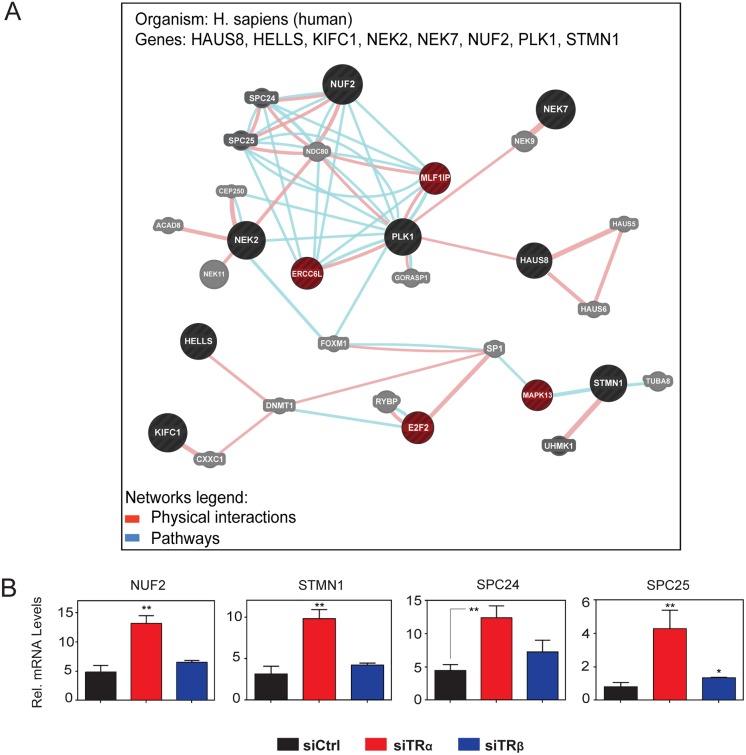
TRα involvement in regulation of formation of mitotic spindle in hADSC. A) Network of interactions among TRα targets involved in mitotic spindle formation, as retrieved by the GeneMania. Circles represent genes and connecting lines represent interactions between genes. GeneMania retrieved known and predicted interactions between these genes and added extra genes that are strongly connected to query genes. (B): qPCR verification of genes identified by GeneMania as part of TRα regulated network. All data are represented as mean ± SD. **, *p* < 0.01; *, *p* < 0.05.

We used GeneMania analysis to highlight relationships of TRα targets associated with specific functional themes defined by IPA (see Fig 7 and additional examples in S12 Fig). For example, analysis of a network generated with eight TRα regulated genes involved in mitotic spindle formation (Fig 7A, black circles) revealed additional TRα regulated genes linked to the network (dark red). Further, independent qRT-PCR analysis of network genes identified yet more TRα repressed targets not detected by the microarray (SPC24, SPC25) (Fig 7B). Both SPC proteins interact with TRα-repressed genes NUF2 and NDC80 to form the NDC80 complex, which is essential for kinetochore/microtubule attachment during cell division. Thus, TRα KD coordinately regulates multiple genes involved in mitotic spindle apparatus, including three members of a crucial kinetochore complex.

**Fig 8:**
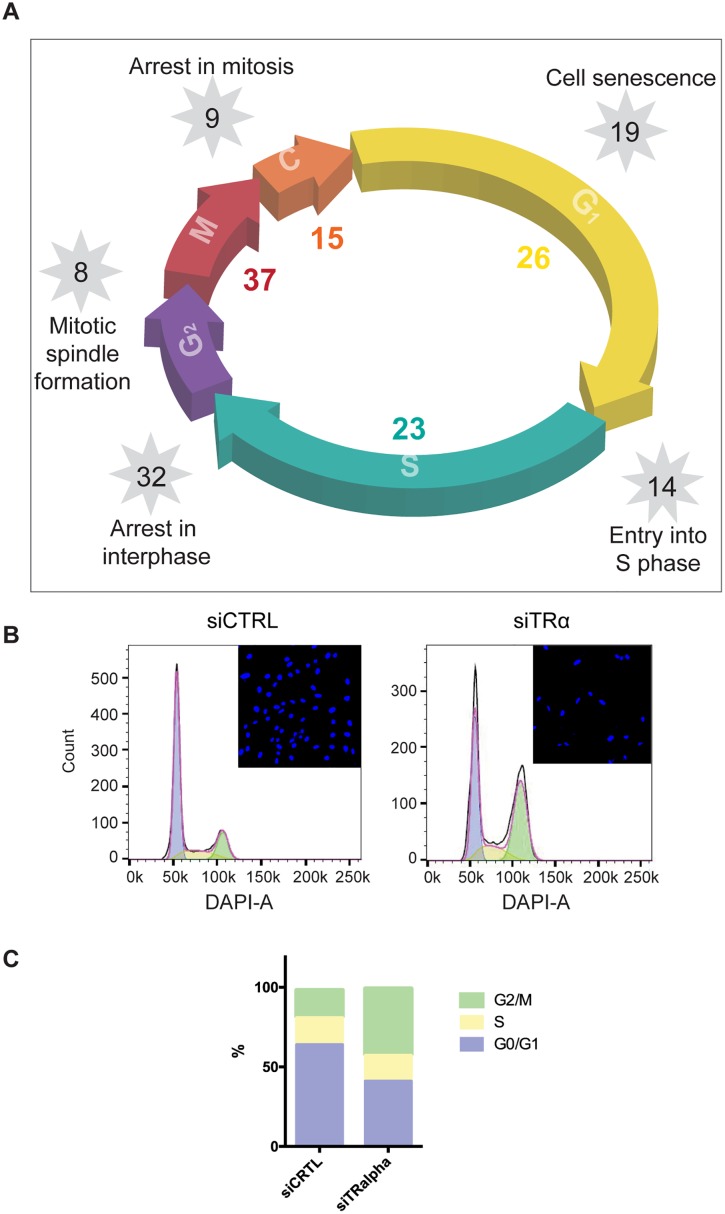
TRα regulates hADSC cell cycle. (A) Schematic of TRα involvement in regulation of cell cycle. (B) Histograms of DNA content in siCtrl and siTRα cells after 6 days siRNA treatment. Inserts: representative images of DAPI stained siCtrl and siTRα hADSC. (C) Stacked columns of cell fractions for each cell-cycle stage (G_0_/G_1_, S and G_2_/M).

Since TRα KD represses multiple genes with roles in many stages of the cell cycle (Table 3, Fig 8A, S8 Table), we assessed effects of TRα KD on cell cycle phases by using DAPI stained cells for flow cytometry based DNA content assessment (Fig 8B and 8C). While control hADSC cells display normal distributions of cells in G0/G1, S and G2/M (Fig 8B and 8C), TRα KD increased numbers of cells in G2/M and reduced numbers of cells in G0/G1 (Fig 8B and 8C). Thus, TRα KD induces G2/M cell cycle arrest, consistent with reduced MI after TRα KD (Table 1).

### TRα1 and TRα2 Regulate Distinct hADSC Genes

**Fig 9:**
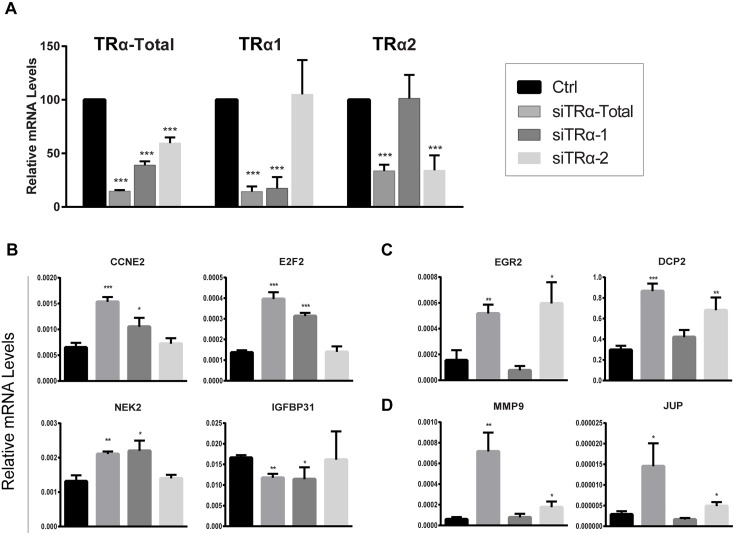
TRα1 and TRα2 KD influences distinct genes in hADSC. (A) Results of qRT-PCR transcript analysis of TRα and TRα1 and TRα2 splice variants after specific siRNA treatment. (B-D) Effects of TRα splice variant KD on TR target genes, B TRα1 specific, C, TRα2 specific, D, genes that require optimal levels of TRα1 and TRα2.

We considered the possibility that TRα-dependent and ligand independent effects on gene expression were mediated by the non-hormone binding TRα2 splice variant. We devised specific siRNA for TRα1 and TRα2 (Materials and Methods) and verified that TRα1 specific siRNA reduced total TRα mRNA and also selectively reduced TRα1 without affecting TRα2 transcripts (Fig 9A). Likewise, TRα2 specific siRNA reduced total TRα levels and selectively reduced TRα2 without affecting TRα1. We detected TRα1 and TRα2-specific changes in TRα target genes after short (2 day) siRNA treatment (Fig 9B, 9C and 9D). One group of transcripts responded selectively to TRα1 KD (Fig 9B). These included CCNE2, E2F2 and NEK2 which were upregulated by total TRα and TRα1 specific KD and unaffected by TRα2 KD. Another group of genes (including EGR2 and DCP2) responded to total TRα KD and TRα2, but were unaffected by TRα1 specific KD (Fig 9C). Finally, some genes responded to total TRα KD, displayed no response to TRα1 KD and were only weakly affected by TRα2 KD, including MMP9 and JUP (Fig 9D). Thus, KD of TRα1 and TRα2 leads to distinct changes in gene expression, both forms of TRα display selective effects on some genes and both may be required for optimal regulation of others.

## Discussion

We began this study with non-biased transcript analysis to search for genes with potential roles in hADSC multipotency or differentiation processes. Our studies revealed that TRα transcripts predominate in hADSC and that both TRα1 and TRα2 are present. By contrast, TRβ1 is expressed at low levels and upregulated during hADSC differentiation. Despite the presence of TRα and TRβ proteins, responses of standard TRE-dependent reporters to T3 after TR transfection and emergence of T3 regulated genes in hADSC-derived differentiated cells, no T3 responses were seen in the parental hADSC. Further investigation revealed that TRs were predominantly extranuclear and siRNA-based KD revealed that both TRs influence hADSC morphology, gene expression and cell division in the absence of hormone. Thus, we propose that extranuclear TRs regulate hADSC biology via processes that involve non-canonical hormone-independent pathways. We have not found analogous examples of other cell types in which TRs do not respond to T3 in the literature, but TH responses are selectively suppressed in hepatocytes in fatty liver without obvious changes in components of the TR signaling machinery [63].

While TRα and TRβ exert similar effects on gene expression in homologous cell types [18–20,64], TRα and TRβ KD led to striking and unique changes in hADSC appearance. TRα KD dependent changes are consistent with disturbed cell cycle progression and G2/M arrest and were accompanied by widespread TRα KD-dependent deregulation (predominantly de-repression) of cell cycle genes. For example, TRα regulates mitotic spindle by repressing components of inner and outer kinetochore [65] as well as proteins involved in kinetochore-microtubule attachment and centrosome duplication and maturation [66] (Figs 7 and 8, Table 3). TRα KD also induced histones, usually only transcribed in S-phase [67] (Figs 7 and 8, Table 3). By contrast, TRβ KD led to changes in cell shape, increases in cell and nuclear size, actin fiber reorganization, a switch to a tubulin based cytoskeletal network, implicated in adult stem cell differentiation [68,69], examples of cells with lipid droplet accumulation and induction of genes associated with differentiation (CIDEA, FABP4, osteopontin; Fig 5, S8 Fig). Thus, TRα plays a crucial permissive role in hADSC cell division processes whereas TRβ1 may be involved in suppression of certain aspects of differentiation. Interestingly, the Privalsky group and our own group previously identified subsets of TR target genes that do not respond to hormone in HepG2 and HeLa cells [18,19]. This gene set, unlike other TR target genes, also displays high degrees of subtype-specificity (see Table 1 in reference [16]). Thus, mechanisms that lead to TR hormone-independent activity may be associated with subtype selectivity.

We think that striking TR subtype selectivity in hADSC emerge from unique TRα and TRβ-dependent effects upon distinct non-genomic pathways [38,39] which, in turn, may be linked to distinct TR locations within the cytoplasm. Both major TRα splice variants colocalize with mitochondria in hADSC, as documented in cardiomyocytes [33], and, to a lesser extent, with ER. TRβ appears evenly distributed through the cytoplasm and does not co-localize with TRα to any appreciable degree. It will be interesting to determine whether TRα-specific effects on hADSC are related to disruption of TRα interactions with mitochondria, well known to influence cell cycle and apoptosis [60].

We cannot eliminate the possibility that some TR activities are related to canonical effects of low levels of nuclear TR on gene expression and, in fact, favor the explanation that concerted effects of TRs in the cytoplasm and nucleus may account for some TR effects on gene expression [34]. We found examples of hADSC TR subtype specific target genes associated with known TR binding sites [20,56]; for example, the TRα-specific JARID2 and E2F2 genes are associated with TRα-specific binding sites whereas the TRα/TRβ regulated FOSL1 locus contains binding sites for both TRs (S13 Fig) [20,56]. Since TRs regulate hADSC genes that encode key regulatory proteins, including the TRα-specific E2F2, involved in stem cell self-renewal [70], and STAT3, required for ESC pluripotent state [71], it is possible that TR subtype-specific changes in small numbers of regulatory proteins could lead to amplified effects on hADSC transcriptional networks.

Why do TRs act in hormone-independent manner in hADSC? The hADSC growth medium does not contain obvious sources of TH-like ligands. While hADSC could produce endogenous TR ligands, we think that it is likelier that TRs do not respond to hormone in this cell type and that unliganded TRs are active. As mentioned, the JARID2, E2F2 and FosL1 genes are repressed by the same TR subtypes in hADSC and HepG2 cells in the absence of hormone [20,56] but nevertheless respond to T3 in HepG2 and in C17.2 cells. This suggests that these genes are similarly regulated by unliganded TRs but unable to respond to T3 in hADSC.

We considered the possibility that the non-hormone binding TRα2 splice variant mediates ligand-independent TR activities. TRα1/TRα2 specific KD indeed revealed target genes that do respond selectively to TRα2 KD (Fig 9). To our knowledge, this is the first time that TRα2 has been shown to exert effects on gene expression that are distinct from its weak inhibitory effects on liganded TRα1 and TRβ1 [31]. There are analogies between TRα2 and a structurally similar estrogen receptor (ER) β splice variant, termed ERβ2 or ERβcx [72,73] which was originally thought to work solely by inhibition of ERα [74] but recently also shown to independently regulate large numbers of genes in prostate cancer cells [75,76]. Our results, however, also reveal that TRα1 and TRβ1 also regulate hADSC genes in ligand-independent fashion in this cell type and we have confirmed that TRα2 KD does not restore the capacity for T3 response at TR targets, such as KLF9, in hADSC (S14 Fig). Thus, we think that it is unlikely that the presence of active TRα2 accounts for all ligand-independent TR actions in hADSC.

We think that it is likely that lack of ligand response is related to the predominantly cytoplasmic localization of TRs in this cell type and, therefore, to factors that promote extranuclear TR actions. Studies to dissect TR actions in hADSC will be difficult; previous studies of non-genomic TR actions have relied on ligands to manipulate TR activity [34] and this approach does not seem feasible in hADSC. There are indications that TRs acquire the capacity to regulate genes in canonical T3-responsive fashion during hADSC differentiation and it may be fruitful to consider roles of gene products that predominate in parental hADSC and TRs. If TRs do prove to regulate some hADSC target genes directly, it may be interesting to consider whether an unusual spectrum of TR interacting proteins is important in ligand-independent activity; TRα1 binds cofactors with roles in cell cycle in a T3-independent fashion [77]. Alternatively, post-translational TR modifications such as SUMOylation, which changes TR-T3 response in a subtype specific manner and is important in preadipocyte differentiation [78], could be important.

Unliganded TRα may also be active in other undifferentiated cell types. TRs are expressed and functional in ESC/iPSC and we detected a switch from TRα to TRβ during ESC/iPSC differentiation along the hepatocyte lineage *in vitro* [44]. Unliganded TRα is also important in preadipocyte differentiation and myoblast proliferation and differentiation [79,80] and early stages of Xenopus metamorphosis [23,25,26]. It will be interesting to ask if these effects also involve actions of predominantly cytoplasmic TRα.

Finally, hADSC display potential uses in regenerative therapies [31,40,41,81]. It is therefore important to understand how TRs act in this cell context and whether manipulation of hADSC TR activities could have medically useful applications. The fact that TRs display ligand-independent actions in hADSC in culture leads us to suspect that TRs may display similar activities in adult stem cells *in vivo* and it will be interesting to determine whether TRα acts in hormone-independent fashion in ADSC pools in animals or humans.

## Supporting Information

S1 Fig Expression of TRα1 and TRα2 during differentiation of hADSC. Expression of TRα1 **(A)** and TRα2 **(B)** during adipogenesis (*A*), chondrogenesis (*C*) and osteogenesis (*O*) was assessed by qPCR.
(TIF)

S2 Fig Absence of T3 response in hADSC. **(A)** Results of qRT-PCR transcript analysis of HR and KLF9 in hADSC and hADSC-derived adipocytes after treatment with vehicle control or 100nM T3 in DMSO. **(B)** Panel shows results of luciferase assays performed on extracts of hADSC that were transfected with reporters containing two copies of each TRE (DR4, F2) and FLAG-tagged TR expression vectors and treated with T3 (100nM) or GC-1 (100nM) for 18h. **(C)** Microarray analysis of gene regulation in cell lineages after T3 treatment (100nM). Microarray data are deposited in the Gene Expression Omnibus; accession number GSE75433.
(TIF)

S3 Fig Subcellular localization of TRα1, TRα2 and TRβ in hADSC. Double-immunofluorescence analysis of TRα1 or TRα2, respectively (green), and TRβ (red): presence, distribution and colocalization. Bar: = 50μm.
(TIF)

S4 Fig Negative control for S3 Fig. The specificity of immunofluorescence was tested by the omission of primary antibodies (**A**). Overlay A and nuclear staining (**B**). Bar: = 100μm.
(TIF)

S5 Fig Efficient knockdown of TR subtypes in hADSC. **(A)** Panels show TR transcript levels after hADSC were transfected with TRα and/or TRβ siRNA at 50 nM final concentration and **(B, C)** TRα1 or TRα2 protein levels assessed by Western blot. We note that TRα2 migrates at a position that is suggestive of higher molecular weight (60KD) than predicted from its primary sequence (50KD), but also note that species of similar size have been noted in previous characterization by western blot, see information in ThermoFisher Scientific catalog.
(TIF)

S6 Fig Effects of TR silencing on immunofluorescence signals. A) Double immunofluorescence analysis of TRα1 or TRα2, respectively (green), and TRβ (red) in siCtrl HADSC: presence, distribution and colocalization; B) After knockdown of TRα1 and TRβ in HADSC, signal is absent, while in TRα2 KD cells signal is reduced. Bar: = 50μm.
(TIF)

S7 Fig Negative control for siRNA knockdown control in S6 Fig. The specificity of immunofluorescence was tested by the omission of primary antibodies. Bar: = 50μm.
(TIF)

S8 Fig Differential effects of TR silencing on cell division. Confocal images of hADSC (A): Ctrl, siTRα and siTRβ showing immunostaining for tubulin α (green) and DNA (nuclei and chromosomes) counterstained with DAPI. Numerous mitosis (yellow arrows) were seen in Ctrl and siTRβ. Binuclear cell (blue arrows). Bars: 50 μm. Image analysis was performed in Zen 2010/ Las AF Lite and Imaris 8.1 software for distribution of nuclear surface area in (B) and cell size (cell surface area) in (C). *compared to control, ***p≤0.001; # siTRα vs. siTRβ, ### p≤0.001.
(TIF)

S9 Fig Overlaps between effects of TR knockdown compared to knockdown of an unrelated TF. Venn diagram of genes regulated by TRα, TRβ or ETV5. Diagrams represent the number of genes regulated after TRα, TRβ or ETV5 knockdown. Microarray data are deposited in the Gene Expression Omnibus (GEO); accession number GSE75692.
(TIF)

S10 Fig TRα and TRβ regulate distinct target genes in hADSC. qPCR verification of TRα and/or TRβ target genes as identified by microarray analysis.
(TIF)

S11 Fig Knockdown of TRα in hADSC. Transcript levels of TRα, NEK2, TP53INP1, STAT3 and ICMT after hADSC were transfected with three different On-TARGET Plus TRα siRNAs at 50 nM final concentration.
(TIF)

S12 Fig TRα involvement in regulation of different aspects of cell cycle regulation in hADSC. Network of interactions among TRα targets involved in endoreduplication (A) and arrest in mitosis (B), as retrieved by GeneMania. Analysis of network generated with TRα regulated genes involved in these processes (large black circles) revealed genes that are strongly connected to query genes including additional TRα regulated genes (red).
(TIF)

S13 Fig Representation of selected TR hADSC target genes that contain verified TR binding regions. TRα (red bar), TRβ (blue bar) and RXR (grey bar) binding sites located 30K bp upstream to 10K bp downstream of Fosl1, Jarid2 and E2f2, as determined by Chatonnet et al. (17) in mouse neural cells (geodataset series GSE38347). TRα, TRβ and RXR peak bed files were downloaded directly from geodatasets GSM940399, GSM940400, and GSM940401, respectively, and uploaded into the UCSC Genome Browser for viewing and image export.
(TIF)

S14 Fig TRα2 knockdown does not result in the induction of T3-responsive gene KLF9. qPCR confirms 50nM siRNA knockdown of TRα2 (A) in hADSCs treated with either DMSO or T3 and shows that KLF9 expression (B) is not significantly changed in either of these conditions.
(TIF)

S1 Table On-TARGET Plus TR siRNA sequences. (DOCX)

S2 Table Transcription Factors and associated partners were identified among the significantly affected genes through comparison to AnimalTFDB 2.0. (DOCX)

S3 Table Change in mRNA expression of NRs after hADSC differentiation. In the Nuclear receptors and coregulators PCR array, fold expression differences between hADSC and differentiated cells were analyzed through the SA Biosciences Web page. All experiments were run in triplicates.
(DOCX)

S4 Table Microarray analysis of gene regulation in adipocytes after T3 treatment. Microarray data are deposited in the Gene Expression Omnibus; accession number GSE75433.
(DOCX)

S5 Table Microarray analysis of gene regulation in chondrocytes after T3 treatment. Microarray data are deposited in the Gene Expression Omnibus; accession number GSE75433.
(DOCX)

S6 Table Microarray analysis of gene regulation in osteocytes after T3 treatment. Microarray data are deposited in the Gene Expression Omnibus; accession number GSE75433.
(DOCX)

S7 Table Differentially expressed genes in the TRα and TRβ-mediated processes obtained from GeneCodis using SlimProcess database (Table 2). (DOCX)

S8 Table List of the differentially expressed genes after TRα or TRβ knockdown in the enriched cell cycle-related functional themes (IPA) in hADSC (Table 3). (DOCX)

## References

[1] Zhang J, Lazar MA. The mechanism of action of thyroid hormones. Annu Rev Physiol 2000;62:439–66. 10.1146/annurev.physiol.62.1.439 10845098

[2] Li D, Yamada T, Wang F, Vulin AI, Samuels HH. Novel roles of retinoid X receptor (RXR) and RXR ligand in dynamically modulating the activity of the thyroid hormone receptor/RXR heterodimer. J Biol Chem 2004;279:7427–37. 10.1074/jbc.M311596200 14668324

[3] Yen PM. Physiological and molecular basis of thyroid hormone action. Physiol Rev 2001;81:1097–142. . 11427693 10.1152/physrev.2001.81.3.1097

[4] Cheng S-Y, Leonard JL, Davis PJ. Molecular aspects of thyroid hormone actions. Title. Endocr Rev 2010;31:139–70. 10.1210/er.2009-0007 20051527 PMC2852208

[5] Astapova I, Hollenberg AN. The in vivo role of nuclear receptor corepressors in thyroid hormone action. Biochim Biophys Acta 2013;1830:3876–81. 10.1016/j.bbagen.2012.07.001 22801336 PMC3529203

[6] Cheng S-Y. Isoform-dependent actions of thyroid hormone nuclear receptors: lessons from knockin mutant mice. Steroids 2005;70:450–4. 10.1016/j.steroids.2005.02.003 15862829

[7] Moran C, Chatterjee K. Resistance to thyroid hormone due to defective thyroid receptor alpha. Best Pract Res Clin Endocrinol Metab 2015;29:647–57. 10.1016/j.beem.2015.07.007 26303090 PMC4559105

[8] Ortiga-Carvalho TM, Sidhaye AR, Wondisford FE. Thyroid hormone receptors and resistance to thyroid hormone disorders. Nat Rev Endocrinol 2014;10:582–91. 10.1038/nrendo.2014.143 25135573 PMC4578869

[9] Baxter JD, Webb P. Thyroid hormone mimetics: potential applications in atherosclerosis, obesity and type 2 diabetes. Nat Rev Drug Discov 2009;8:308–20. 10.1038/nrd2830 19337272

[10] Brent GA. Mechanisms of thyroid hormone action. J Clin Invest 2012;122:3035–43. 10.1172/JCI60047 22945636 PMC3433956

[11] Kim WG, Cheng S. Thyroid hormone receptors and cancer. Biochim Biophys Acta 2013;1830:3928–36. 10.1016/j.bbagen.2012.04.002 22507269 PMC3406244

[12] Aranda A, Martinez-Iglesias O, Ruiz-Llorente L, Garcia-Carpizo V, Zambrano A. Thyroid receptor: roles in cancer. Trends Endocrinol Metab 2009;20:318–24. 10.1016/j.tem.2009.03.011 19716314

[13] Cheng S-Y. Thyroid hormone receptor mutations in cancer. Mol Cell Endocrinol 2003;213:23–30. 10.1016/j.mce.2003.10.051 15062571

[14] Andersson ML, Nordström K, Demczuk S, Harbers M, Vennström B. Thyroid hormone alters the DNA binding properties of chicken thyroid hormone receptors alpha and beta. Nucleic Acids Res 1992;20:4803–10. 1408794 10.1093/nar/20.18.4803 PMC334235

[15] Darling DS, Carter RL, Yen PM, Welborn JM, Chin WW, Umeda PK. Different dimerization activities of alpha and beta thyroid hormone receptor isoforms. J Biol Chem 1993;268:10221–7. 7683671

[16] Harbers M, Wahlström GM, Vennström B. Transactivation by the thyroid hormone receptor is dependent on the spacer sequence in hormone response elements containing directly repeated half-sites. Nucleic Acids Res 1996;24:2252–9. 10.1093/nar/24.12.2252 8710493 PMC145925

[17] Yen PM, Feng X, Flamant F, Chen Y, Walker RL, Weiss RE, et al Effects of ligand and thyroid hormone receptor isoforms on hepatic gene expression profiles of thyroid hormone receptor knockout mice. EMBO Rep 2003;4:581–7. 10.1038/sj.embor.embor862 12776178 PMC1319202

[18] Lin JZ, Sieglaff DH, Yuan C, Su J, Arumanayagam AS, Firouzbakht S, et al Gene Specific Actions of Thyroid Hormone Receptor Subtypes. PLoS One 2013;8:e52407 10.1371/journal.pone.0052407 23300972 PMC3536777

[19] Chan IH, Privalsky ML. Isoform-Specific Transcriptional Activity of Overlapping Target Genes that Respond to Thyroid Hormone Receptors α1 and β1. Mol Endocrinol 2009;23:1758–75. 10.1210/me.2009-0025 19628582 PMC2775939

[20] Chatonnet F, Guyot R, Benoît G, Flamant F. Genome-wide analysis of thyroid hormone receptors shared and specific functions in neural cells. Proc Natl Acad Sci U S A 2013;110:E766–75. 10.1073/pnas.1210626110 23382204 PMC3581916

[21] Feng X, Jiang Y, Meltzer P, Yen PM. Thyroid hormone regulation of hepatic genes in vivo detected by complementary DNA microarray. Mol Endocrinol 2000;14:947–55. 10.1210/mend.14.7.0470 10894146

[22] Mittag J, Wallis K, Vennstrom B. Physiological consequences of the TRalpha1 aporeceptor state. Heart Fail Rev 2010;15:111–5. 10.1007/s10741-008-9119-5 19009345

[23] Bernal J, Morte B. Thyroid hormone receptor activity in the absence of ligand: Physiological and developmental implications. Biochim Biophys Acta—Gen Subj 2013;1830:3893–9. 10.1016/j.bbagen.2012.04.014 22554916

[24] Hu X, Lazar M a. Transcriptional repression by nuclear hormone receptors. Trends Endocrinol Metab 2000;11:6–10. S1043-2760(99)00215-5 [pii]. 10.1016/S1043-2760(99)00215-5 10652499

[25] Wen L, Shi Y-B. Regulation of growth rate and developmental timing by Xenopus thyroid hormone receptor α. Dev Growth Differ 2015;58:1–9. 10.1111/dgd.12231 26219216 PMC6296368

[26] Choi J, Suzuki K-IT, Sakuma T, Shewade L, Yamamoto T, Buchholz DR. Unliganded thyroid hormone receptor alpha regulates developmental timing via gene repression in Xenopus tropicalis. Endocrinology 2015;156:735–44. 10.1210/en.2014-1554 25456067 PMC4298327

[27] Flamant F, Poguet A-L, Plateroti M, Chassande O, Gauthier K, Streichenberger N, et al Congenital hypothyroid Pax8(-/-) mutant mice can be rescued by inactivating the TRalpha gene. Mol Endocrinol 2002;16:24–32. 10.1210/mend.16.1.0766 11773436

[28] Wen L, Shi YB. Unliganded thyroid hormone receptor α Controls developmental timing in Xenopus tropicalis. Endocrinology 2015;156:721–34. 10.1210/en.2014-1439 25456066 PMC4298314

[29] Pasca di Magliano M, Di Lauro R, Zannini M. Pax8 has a key role in thyroid cell differentiation. Proc Natl Acad Sci U S A 2000;97:13144–9. 10.1073/pnas.240336397 11069301 PMC27192

[30] Mansouri A, Chowdhury K, Gruss P. Follicular cells of the thyroid gland require Pax8 gene function. Nat Genet 1998;19:87–90. 10.1038/ng0598-87 9590297

[31] Williams GR. Thyroid Hormone Actions in Cartilage and Bone. Eur Thyroid J 2012;2:3–13. 10.1159/000345548 24783033 PMC3821494

[32] Ng L, Kelley MW, Forrest D. Making sense with thyroid hormone—the role of T(3) in auditory development. Nat Rev Endocrinol 2013;9:296–307. 10.1038/nrendo.2013.58 23529044

[33] Xu B, Koenig RJ. Regulation of thyroid hormone receptor alpha2 RNA binding and subcellular localization by phosphorylation. Mol Cell Endocrinol 2005;245:147–57. 10.1016/j.mce.2005.11.010 16356627

[34] Farwell AP, Leonard JL. Nongenomic actions of thyroid hormone during fetal brain development. Curr Opin Endocrinol Diabetes 2005;12:17–22. 10.1097/01.med.0000152036.70617.1e

[35] Flamant F, Gauthier K. Thyroid hormone receptors: the challenge of elucidating isotype-specific functions and cell-specific response. Biochim Biophys Acta 2013;1830:3900–7. 10.1016/j.bbagen.2012.06.003 22704954

[36] Fugier C, Tousaint J-J, Prieur X, Plateroti M, Samarut J, Delerive P. The lipoprotein lipase inhibitor ANGPTL3 is negatively regulated by thyroid hormone. J Biol Chem 2006;281:11553–9. 10.1074/jbc.M512554200 16505486

[37] Chiamolera MI, Sidhaye AR, Matsumoto S, He Q, Hashimoto K, Ortiga-Carvalho TM, et al Fundamentally distinct roles of thyroid hormone receptor isoforms in a thyrotroph cell line are due to differential DNA binding. Mol Endocrinol 2012;26:926–39. 10.1210/me.2011-1290 22570333 PMC3355539

[38] Davis PJ, Goglia F, Leonard JL. Nongenomic actions of thyroid hormone. Nat Rev Endocrinol 2016;12:111–21. 10.1038/nrendo.2015.205 26668118

[39] Gauthier K, Flamant F. Nongenomic, TRbeta-dependent, thyroid hormone response gets genetic support. Endocrinology 2014;155:3206–9. 10.1210/en.2014-1597 25152174

[40] Xu Y, Malladi P, Wagner DR, Longaker MT. Adipose-derived mesenchymal cells as a potential cell source for skeletal regeneration. Curr Opin Mol Ther 2005;7:300–5. 16121695

[41] Zuk PA, Zhu M, Mizuno H. Multilineage cells from human adipose tissue: implications for cell-based therapies. Tissue Eng 2001;7:211–28. 10.1089/107632701300062859 11304456

[42] Obregon M-J. Adipose tissues and thyroid hormones. Front Physiol 2014;5:479 10.3389/fphys.2014.00479 25566082 PMC4263094

[43] Williams GR. Thyroid Hormone Actions in Cartilage and Bone. Eur Thyroid J 2013;2:3–13. 10.1159/000345548 24783033 PMC3821494

[44] Cvoro A, Devito L, Milton FA, Noli L, Zhang A, Filippi C, et al A Thyroid hormone receptor/KLF9 axis in human hepatocytes and pluripotent stem cells. Stem Cells 2015;33:416–28. 10.1002/stem.1875 25330987 PMC6317531

[45] Du P, Kibbe WA, Lin SM. lumi: a pipeline for processing Illumina microarray. Bioinformatics 2008;24:1547–8. 10.1093/bioinformatics/btn224 18467348

[46] Smyth GK. Linear models and empirical bayes methods for assessing differential expression in microarray experiments. Stat Appl Genet Mol Biol 2004;3:Article3. 10.2202/1544-6115.1027 16646809

[47] R Development Core Team. R: A language and environment for statistical computing. Vienna, Austria: R Foundation for Statistical Computing; 2009.

[48] Benjamini Yoav H Y. Controlling the False Discovery Rate: A Practical and Powerful Approach to Multiple Testing. J R Stat Soc Ser B 1995;57:289–300.

[49] Zhang HM, Liu T, Liu CJ, Song S, Zhang X, Liu W, et al AnimalTFDB 2.0: A resource for expression, prediction and functional study of animal transcription factors. Nucleic Acids Res 2015;43:D76–81. 10.1093/nar/gku887 25262351 PMC4384004

[50] Tabas-Madrid D, Nogales-Cadenas R, Pascual-Montano A. GeneCodis3: A non-redundant and modular enrichment analysis tool for functional genomics. Nucleic Acids Res 2012;40:W478–83. 10.1093/nar/gks402 22573175 PMC3394297

[51] Nogales-Cadenas R, Carmona-Saez P, Vazquez M, Vicente C, Yang X, Tirado F, et al GeneCodis: Interpreting gene lists through enrichment analysis and integration of diverse biological information. Nucleic Acids Res 2009;37:W317–22. 10.1093/nar/gkp416 19465387 PMC2703901

[52] Carmona-Saez P, Chagoyen M, Tirado F, Carazo JM, Pascual-Montano A. GENECODIS: a web-based tool for finding significant concurrent annotations in gene lists. Genome Biol 2007;8:R3 10.1186/gb-2007-8-1-r3 17204154 PMC1839127

[53] Mostafavi S, Ray D, Warde-Farley D, Grouios C, Morris Q. GeneMANIA: a real-time multiple association network integration algorithm for predicting gene function. Genome Biol 2008;9 Suppl 1:S4 10.1186/gb-2008-9-s1-s4 18613948 PMC2447538

[54] Warde-Farley D, Donaldson SL, Comes O, Zuberi K, Badrawi R, Chao P, et al The GeneMANIA prediction server: Biological network integration for gene prioritization and predicting gene function. Nucleic Acids Res 2010;38:W214–20. 10.1093/nar/gkq537 20576703 PMC2896186

[55] Zuberi K, Franz M, Rodriguez H, Montojo J, Lopes CT, Bader GD, et al GeneMANIA prediction server 2013 update. Nucleic Acids Res 2013;41:W115–22. 10.1093/nar/gkt533 23794635 PMC3692113

[56] Ayers S, Switnicki MP, Angajala A, Lammel J, Arumanayagam AS, Webb P. Genome-wide binding patterns of thyroid hormone receptor beta. PLoS One 2014;9:e81186 10.1371/journal.pone.0081186 24558356 PMC3928038

[57] Zhang A, Sieglaff DH, York JP, Suh JH, Ayers SD, Winnier GE, et al Thyroid hormone receptor regulates most genes independently of fibroblast growth factor 21 in liver. J Endocrinol 2015;224:289–301. 10.1530/JOE-14-0440 25501997

[58] Lammel Lindemann JA, Angajala A, Engler DA, Webb P, Ayers SD. Thyroid hormone induction of human cholesterol 7 alpha-hydroxylase (Cyp7a1) in vitro. Mol Cell Endocrinol 2014;388:32–40. 10.1016/j.mce.2014.02.003 24582860 PMC4180720

[59] Iglesias P, Bayon C, Mendez J, Gancedo PG, Grande C, Diez JJ. Serum insulin-like growth factor type 1, insulin-like growth factor-binding protein-1, and insulin-like growth factor-binding protein-3 concentrations in patients with thyroid dysfunction. Thyroid 2001;11:1043–8. 10.1089/105072501753271734 11762714

[60] Psarra AMG, Sekeris CE. Steroid and thyroid hormone receptors in mitochondria. IUBMB Life 2008;60:210–23. 10.1002/iub.37 18344181

[61] Jackson AL, Burchard J, Leake D, Reynolds A, Schelter J, Guo J, et al Position-specific chemical modification of siRNAs reduces “off-target” transcript silencing. RNA 2006;12:1197–205. 10.1261/rna.30706 16682562 PMC1484422

[62] Yuan C, Lin JZH, Sieglaff DH, Ayers SD, Denoto-Reynolds F, Baxter JD, et al Identical gene regulation patterns of T3 and selective thyroid hormone receptor modulator GC-1. Endocrinology 2012;153:501–11. 10.1210/en.2011-1325 22067320 PMC3249679

[63] Pihlajamäki J, Boes T, Kim E-Y, Dearie F, Kim BW, Schroeder J, et al Thyroid hormone-related regulation of gene expression in human fatty liver. J Clin Endocrinol Metab 2009;94:3521–9. 10.1210/jc.2009-0212 19549744 PMC2741713

[64] Brent GA. Mechanisms of thyroid hormone action. J Clin Invest 2012;122:3035–43. 10.1172/JCI60047 22945636 PMC3433956

[65] Jia L, Kim S, Yu H. Tracking spindle checkpoint signals from kinetochores to APC/C. Trends Biochem Sci 2013;38:302–11. 10.1016/j.tibs.2013.03.004 23598156

[66] Mardin BR, Schiebel E. Breaking the ties that bind: New advances in centrosome biology. J Cell Biol 2012;197:11–8. 10.1083/jcb.201108006 22472437 PMC3317805

[67] Ewen ME. Where the cell cycle and histones meet. Genes Dev 2000;14:2265–70. 10.1101/gad.842100 10995383

[68] Rodriguez JP, Gonzalez M, Rios S, Cambiazo V. Cytoskeletal organization of human mesenchymal stem cells (MSC) changes during their osteogenic differentiation. J Cell Biochem 2004;93:721–31. 10.1002/jcb.20234 15660416

[69] Yourek G, Hussain MA, Mao JJ. Cytoskeletal changes of mesenchymal stem cells during differentiation. ASAIO J 2007;53:219–28. 10.1097/MAT.0b013e31802deb2d 17413564 PMC4035052

[70] Becker KA, Stein JL, Lian JB, van Wijnen AJ, Stein GS. Establishment of histone gene regulation and cell cycle checkpoint control in human embryonic stem cells. J Cell Physiol 2007;210:517–26. 10.1002/jcp.20903 17096384

[71] Raz R, Lee CK, Cannizzaro L a, D’Eustachio P, Levy DE. Essential role of STAT3 for embryonic stem cell pluripotency. Proc Natl Acad Sci U S A 1999;96:2846–51. 10.1073/pnas.96.6.2846 10077599 PMC15857

[72] Leung Y-K, Mak P, Hassan S, Ho S-M. Estrogen receptor (ER)-beta isoforms: a key to understanding ER-beta signaling. Proc Natl Acad Sci U S A 2006;103:13162–7. 10.1073/pnas.0605676103 16938840 PMC1552044

[73] Huang B, Omoto Y, Iwase H, Yamashita H, Toyama T, Coombes RC, et al Differential expression of estrogen receptor alpha, beta1, and beta2 in lobular and ductal breast cancer. Proc Natl Acad Sci U S A 2014;111:1933–8. 10.1073/pnas.1323719111 24449868 PMC3918808

[74] Zhao C, Matthews J, Tujague M, Wan J, Strom A, Toresson G, et al Estrogen receptor beta2 negatively regulates the transactivation of estrogen receptor alpha in human breast cancer cells. Cancer Res 2007;67:3955–62. 10.1158/0008-5472.CAN-06-3505 17440111

[75] Dey P, Jonsson P, Hartman J, Williams C, Strom A, Gustafsson J-A. Estrogen receptors beta1 and beta2 have opposing roles in regulating proliferation and bone metastasis genes in the prostate cancer cell line PC3. Mol Endocrinol 2012;26:1991–2003. 10.1210/me.2012.1227 23028063 PMC3517717

[76] Dey P, Velazquez-Villegas LA, Faria M, Turner A, Jonsson P, Webb P, et al Estrogen Receptor beta2 Induces Hypoxia Signature of Gene Expression by Stabilizing HIF-1alpha in Prostate Cancer. PLoS One 2015;10:e0128239 10.1371/journal.pone.0128239 26010887 PMC4444278

[77] Hahm JB, Schroeder AC, Privalsky ML. The two major isoforms of thyroid hormone receptor, TRalpha1 and TRbeta1, preferentially partner with distinct panels of auxiliary proteins. Mol Cell Endocrinol 2014;383:80–95. 10.1016/j.mce.2013.11.015 24325866

[78] Liu Y-Y, Kogai T, Schultz JJ, Mody K, Brent GA. Thyroid hormone receptor isoform-specific modification by small ubiquitin-like modifier (SUMO) modulates thyroid hormone-dependent gene regulation. J Biol Chem 2012;287:36499–508. 10.1074/jbc.M112.344317 22930759 PMC3476315

[79] Liu YY, Ayers S, Milanesi A, Teng X, Rabi S, Akiba Y, et al Thyroid hormone receptor sumoylation is required for preadipocyte differentiation and proliferation. J Biol Chem 2015;290:7402–15. 10.1074/jbc.M114.600312 25572392 PMC4367250

[80] Milanesi A, Lee J-W, Kim N-H, Liu Y-Y, Yang A, Sedrakyan S, et al Thyroid Hormone Receptor α Plays an Essential Role in Male Skeletal Muscle Myoblast Proliferation, Differentiation and Response to Injury. Endocrinology 2015;157:en20151443 10.1210/en.2015-1443 26451739 PMC4701883

[81] Obregon M-J. Adipose tissues and thyroid hormones. Front Physiol 2014;5:479 10.3389/fphys.2014.00479 25566082 PMC4263094

