# Supplementary figures and images for: Ligand Independent and Subtype-Selective Actions of Thyroid Hormone Receptors in Human Adipose Derived Stem Cells
Source: PLoS One. 2016 Oct 12;11(10):e0164407. doi: 10.1371/journal.pone.0164407 (PMC5061422; doi:10.1371/journal.pone.0164407)

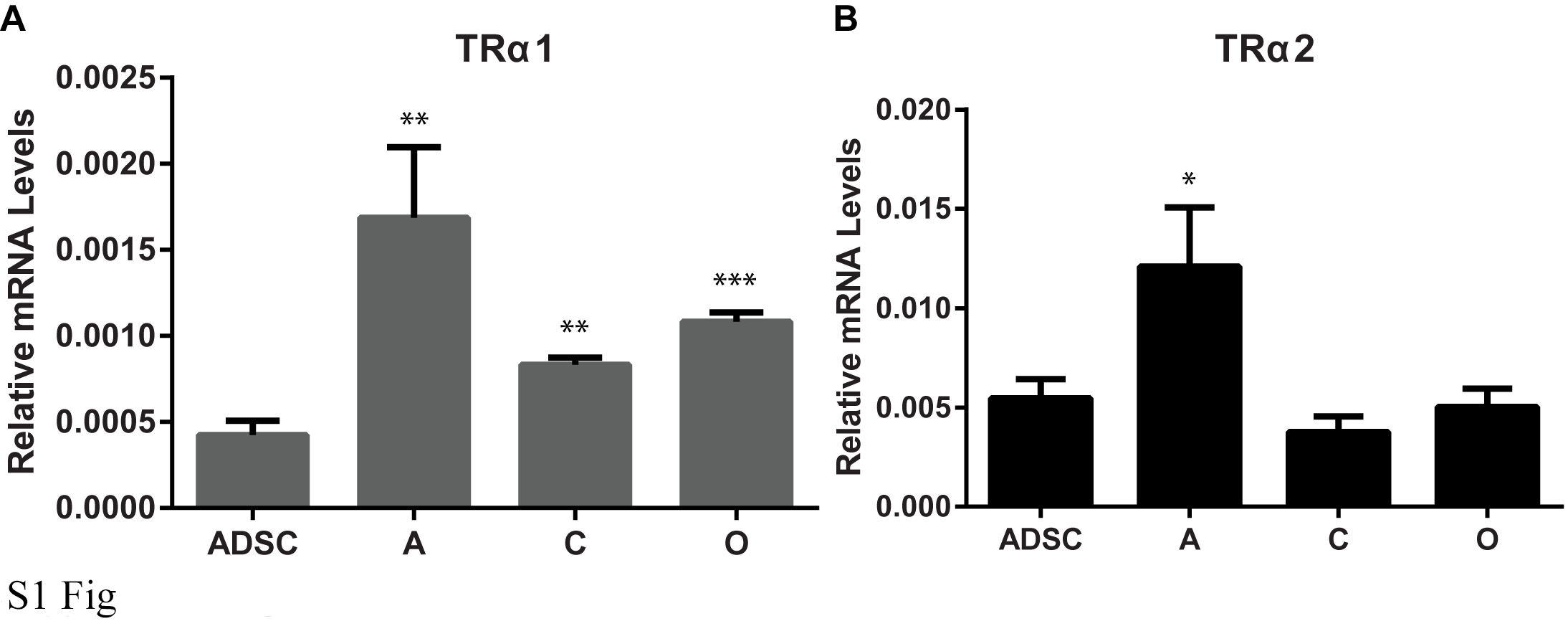

Supplement: S1 Fig — Expression of TRα1 (A) and TRα2 (B) during adipogenesis (A), chondrogenesis (C) and osteogenesis (O) was assessed by qPCR. (TIF) [file pone.0164407.s001.tif]

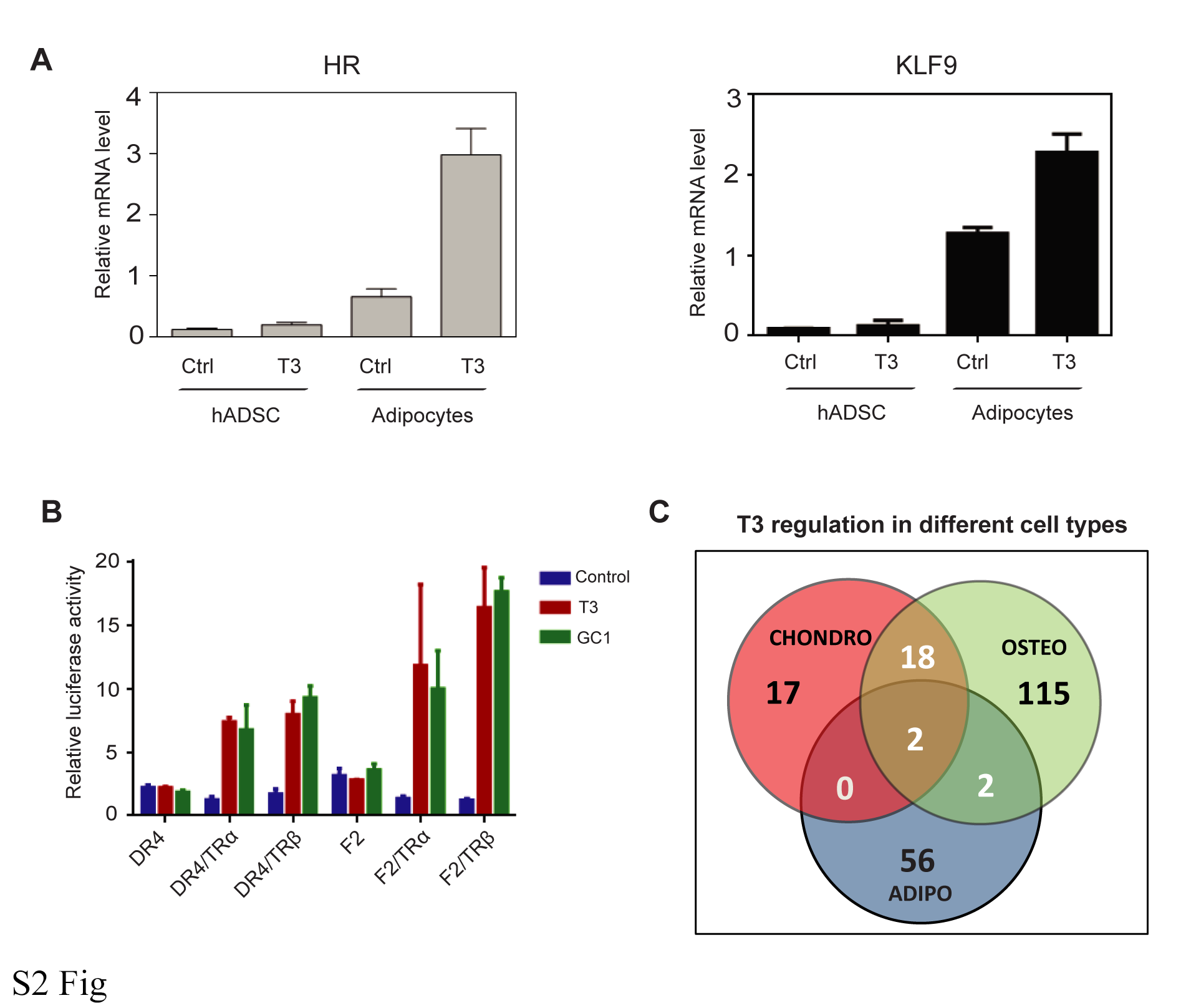

Supplement: S2 Fig — (A) Results of qRT-PCR transcript analysis of HR and KLF9 in hADSC and hADSC-derived adipocytes after treatment with vehicle control or 100nM T3 in DMSO. (B) Panel shows results of luciferase assays performed on extracts of hADSC that were transfected with reporters containing two copies of each TRE (DR4, F2) and FLAG-tagged TR expression vectors and treated with T3 (100nM) or GC-1 (100nM) for 18h. (C) Microarray analysis of gene regulation in cell lineages after T3 treatment (100nM). Microarray data are deposited in the Gene Expression Omnibus; accession number GSE75433. (TIF) [file pone.0164407.s002.tif]

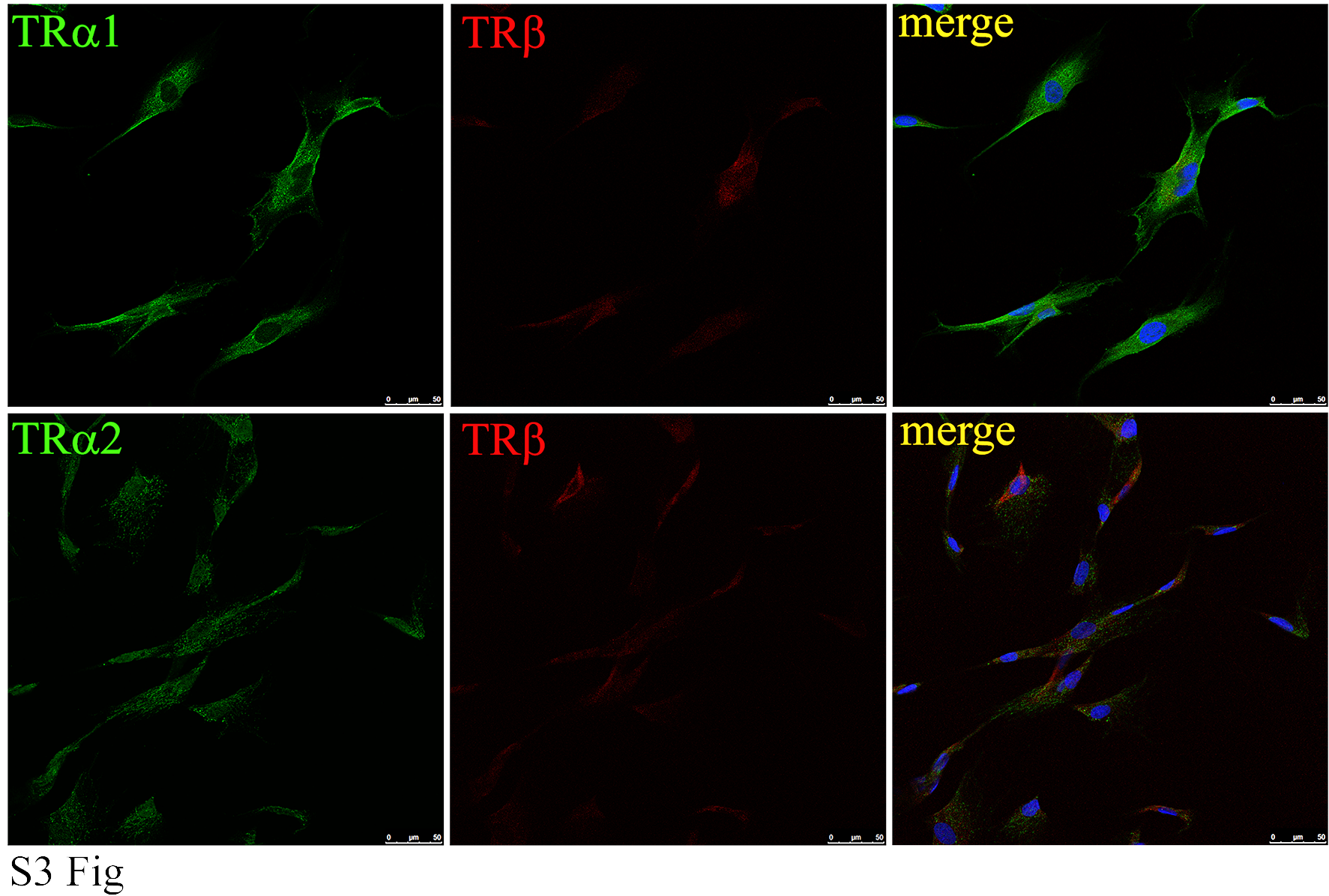

Supplement: S3 Fig — Double-immunofluorescence analysis of TRα1 or TRα2, respectively (green), and TRβ (red): presence, distribution and colocalization. Bar: = 50μm. (TIF) [file pone.0164407.s003.tif]

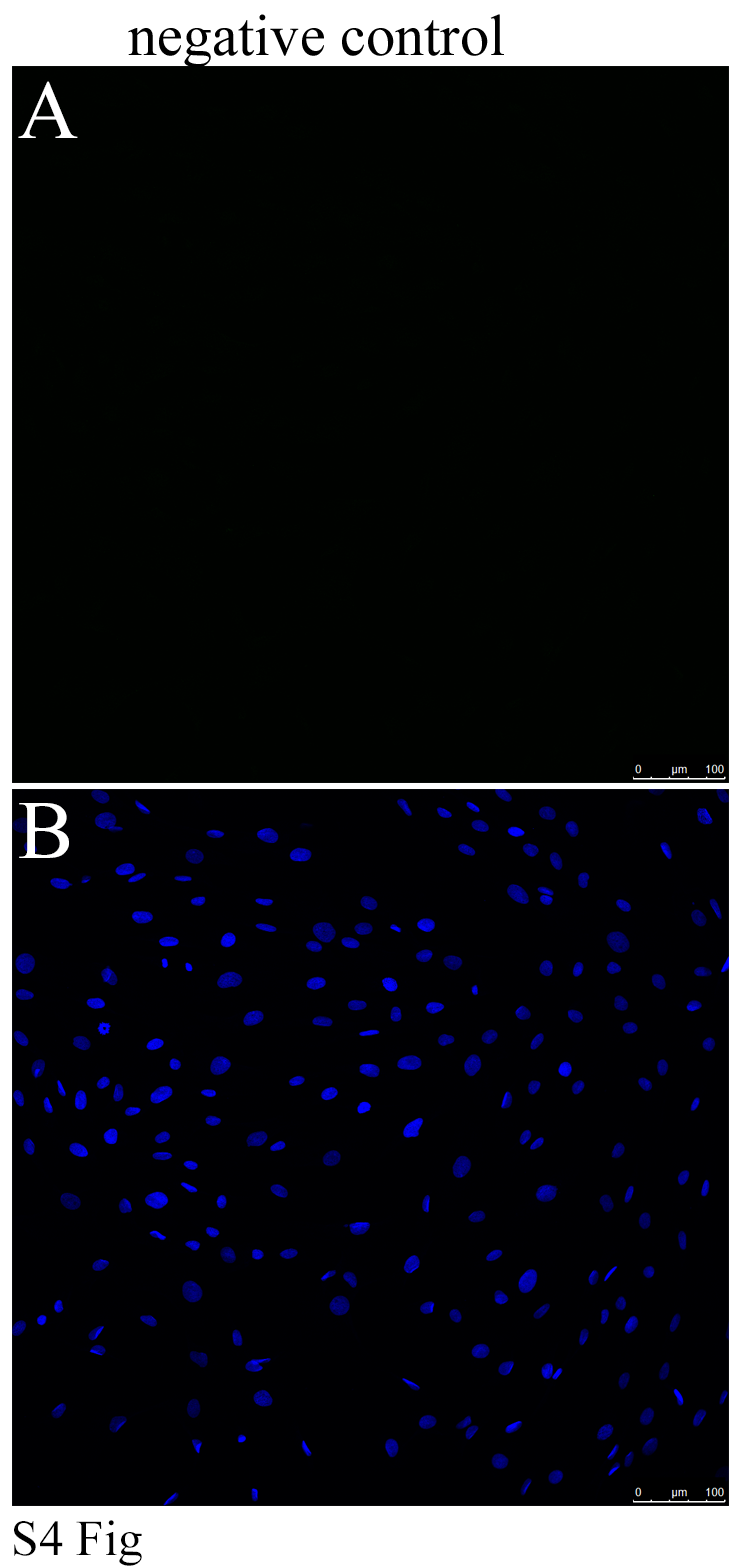

Supplement: S4 Fig — The specificity of immunofluorescence was tested by the omission of primary antibodies (A). Overlay A and nuclear staining (B). Bar: = 100μm. (TIF) [file pone.0164407.s004.tif]

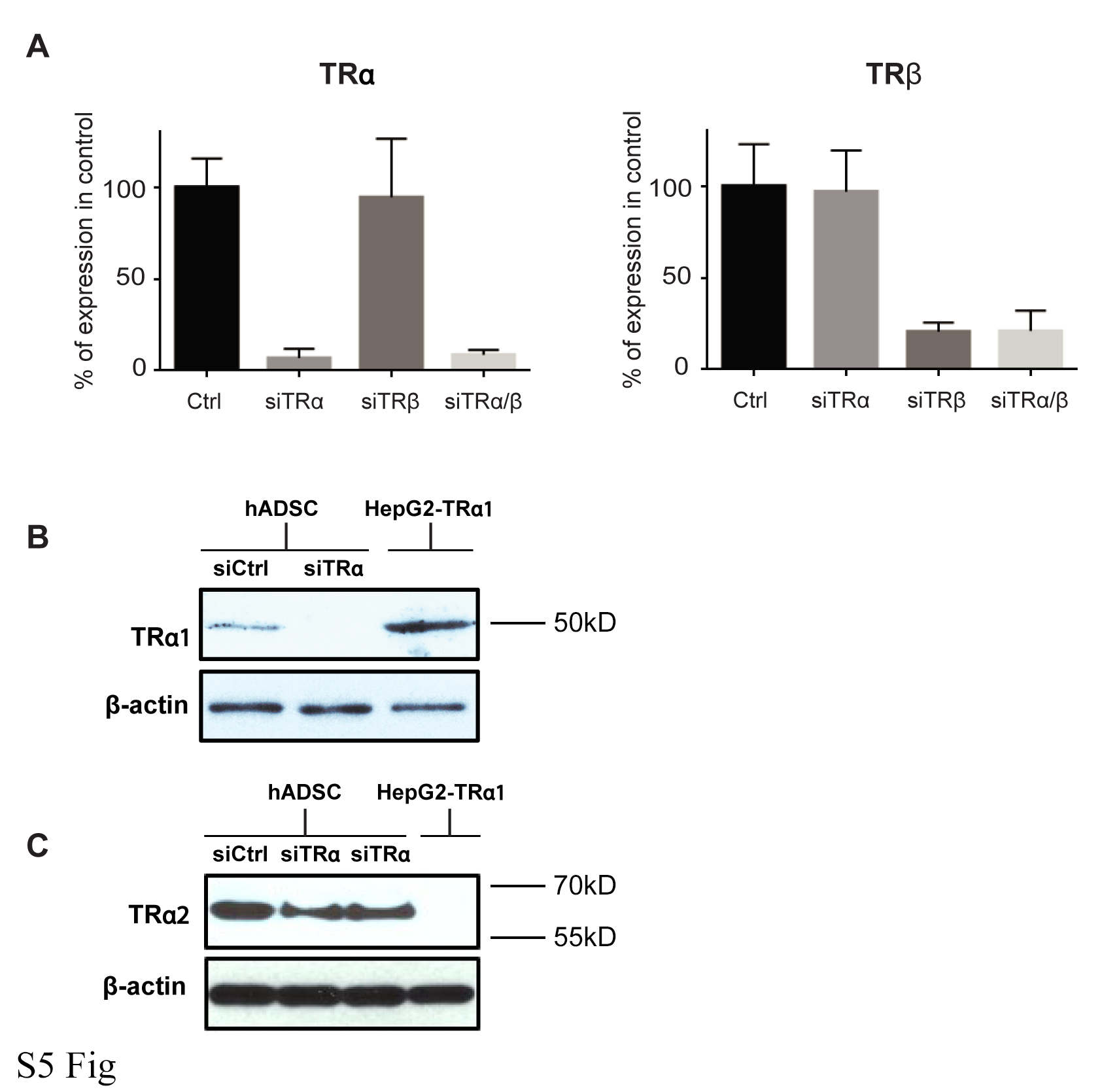

Supplement: S5 Fig — (A) Panels show TR transcript levels after hADSC were transfected with TRα and/or TRβ siRNA at 50 nM final concentration and (B, C) TRα1 or TRα2 protein levels assessed by Western blot. We note that TRα2 migrates at a position that is suggestive of higher molecular weight (60KD) than predicted from its primary sequence (50KD), but also note that species of similar size have been noted in previous characterization by western blot, see information in ThermoFisher Scientific catalog. (TIF) [file pone.0164407.s005.tif]

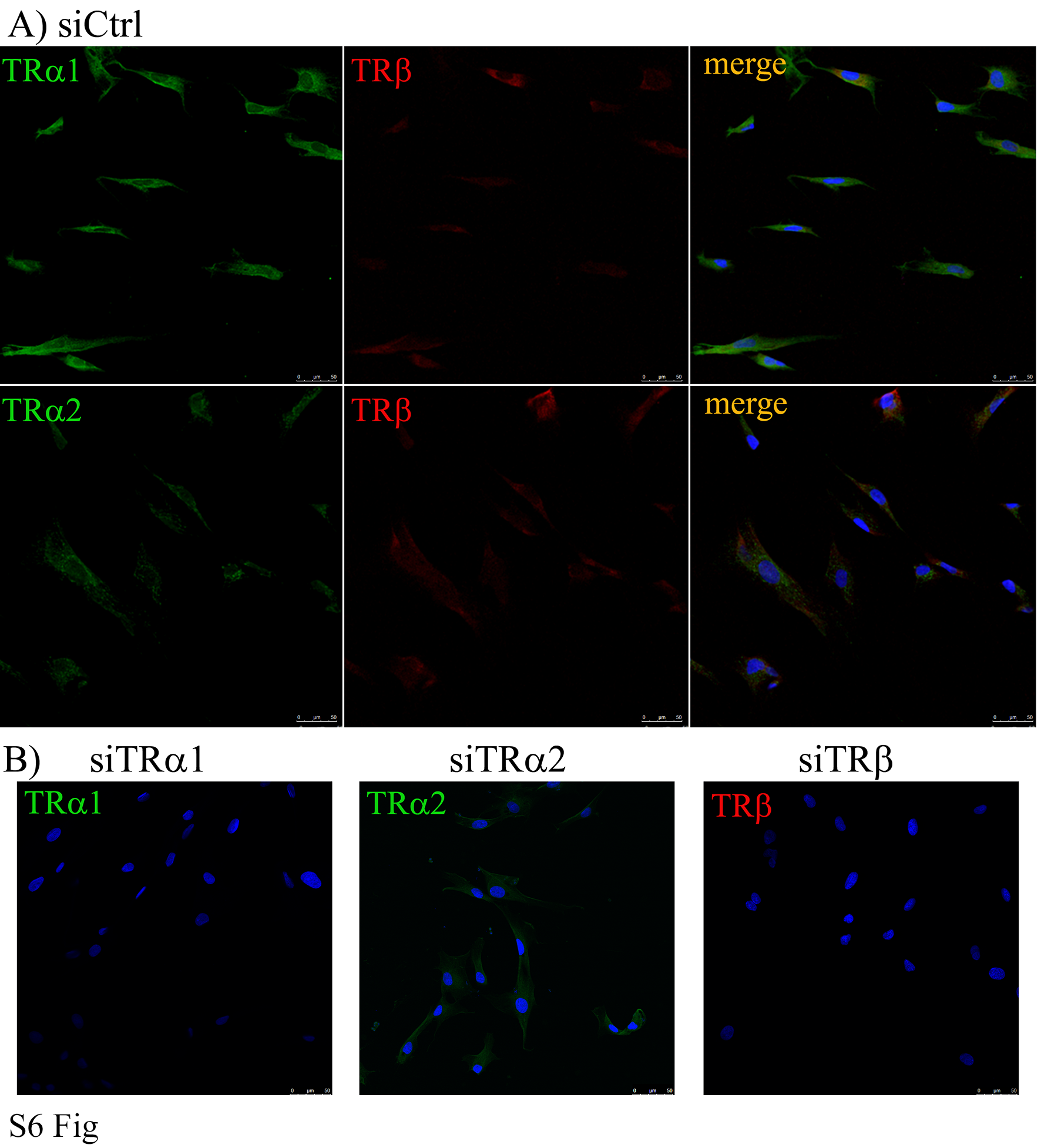

Supplement: S6 Fig — A) Double immunofluorescence analysis of TRα1 or TRα2, respectively (green), and TRβ (red) in siCtrl HADSC: presence, distribution and colocalization; B) After knockdown of TRα1 and TRβ in HADSC, signal is absent, while in TRα2 KD cells signal is reduced. Bar: = 50μm. (TIF) [file pone.0164407.s006.tif]

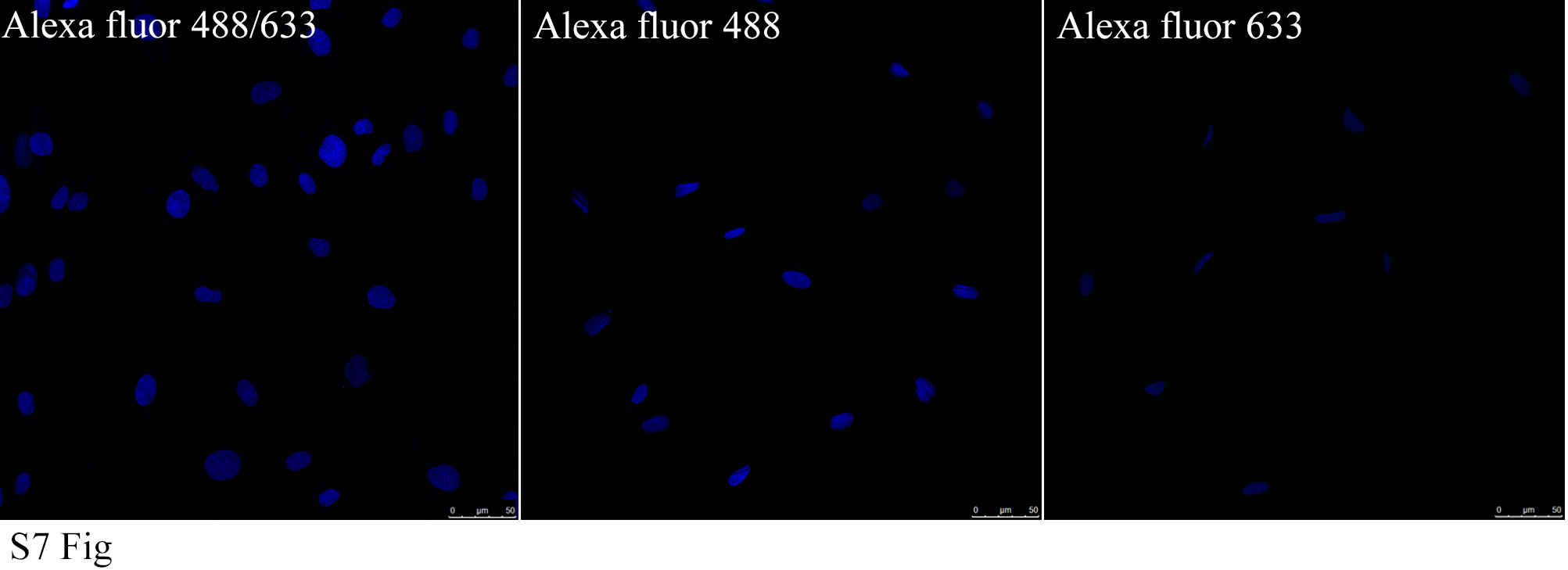

Supplement: S7 Fig — The specificity of immunofluorescence was tested by the omission of primary antibodies. Bar: = 50μm. (TIF) [file pone.0164407.s007.tif]

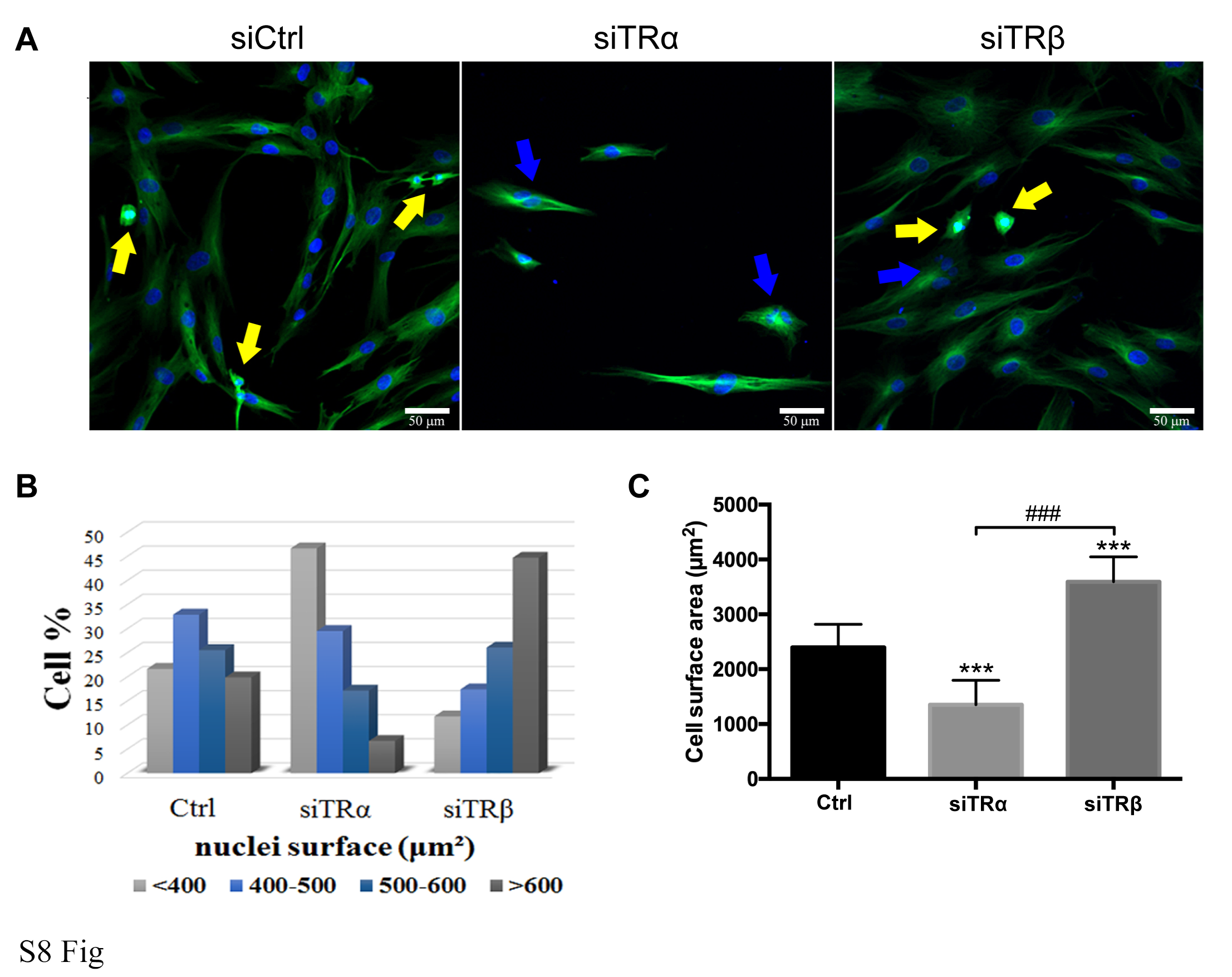

Supplement: S8 Fig — Confocal images of hADSC (A): Ctrl, siTRα and siTRβ showing immunostaining for tubulin α (green) and DNA (nuclei and chromosomes) counterstained with DAPI. Numerous mitosis (yellow arrows) were seen in Ctrl and siTRβ. Binuclear cell (blue arrows). Bars: 50 μm. Image analysis was performed in Zen 2010/ Las AF Lite and Imaris 8.1 software for distribution of nuclear surface area in (B) and cell size (cell surface area) in (C). *compared to control, ***p≤0.001; # siTRα vs. siTRβ, ### p≤0.001. (TIF) [file pone.0164407.s008.tif]

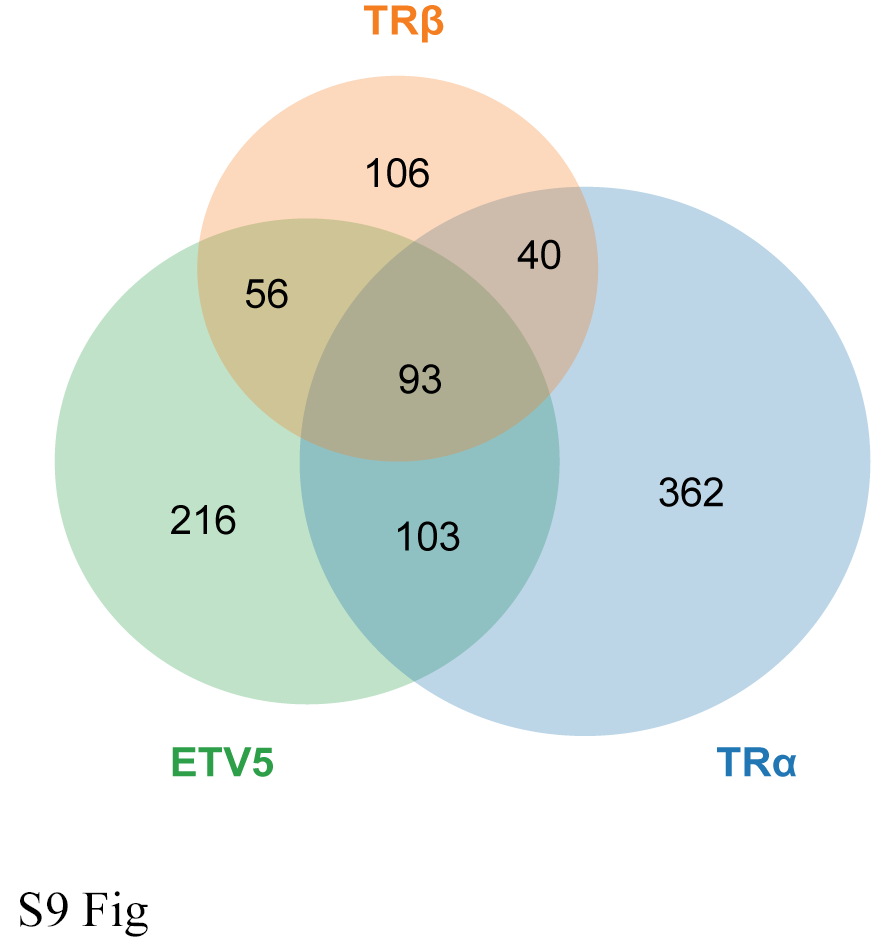

Supplement: S9 Fig — Venn diagram of genes regulated by TRα, TRβ or ETV5. Diagrams represent the number of genes regulated after TRα, TRβ or ETV5 knockdown. Microarray data are deposited in the Gene Expression Omnibus (GEO); accession number GSE75692. (TIF) [file pone.0164407.s009.tif]

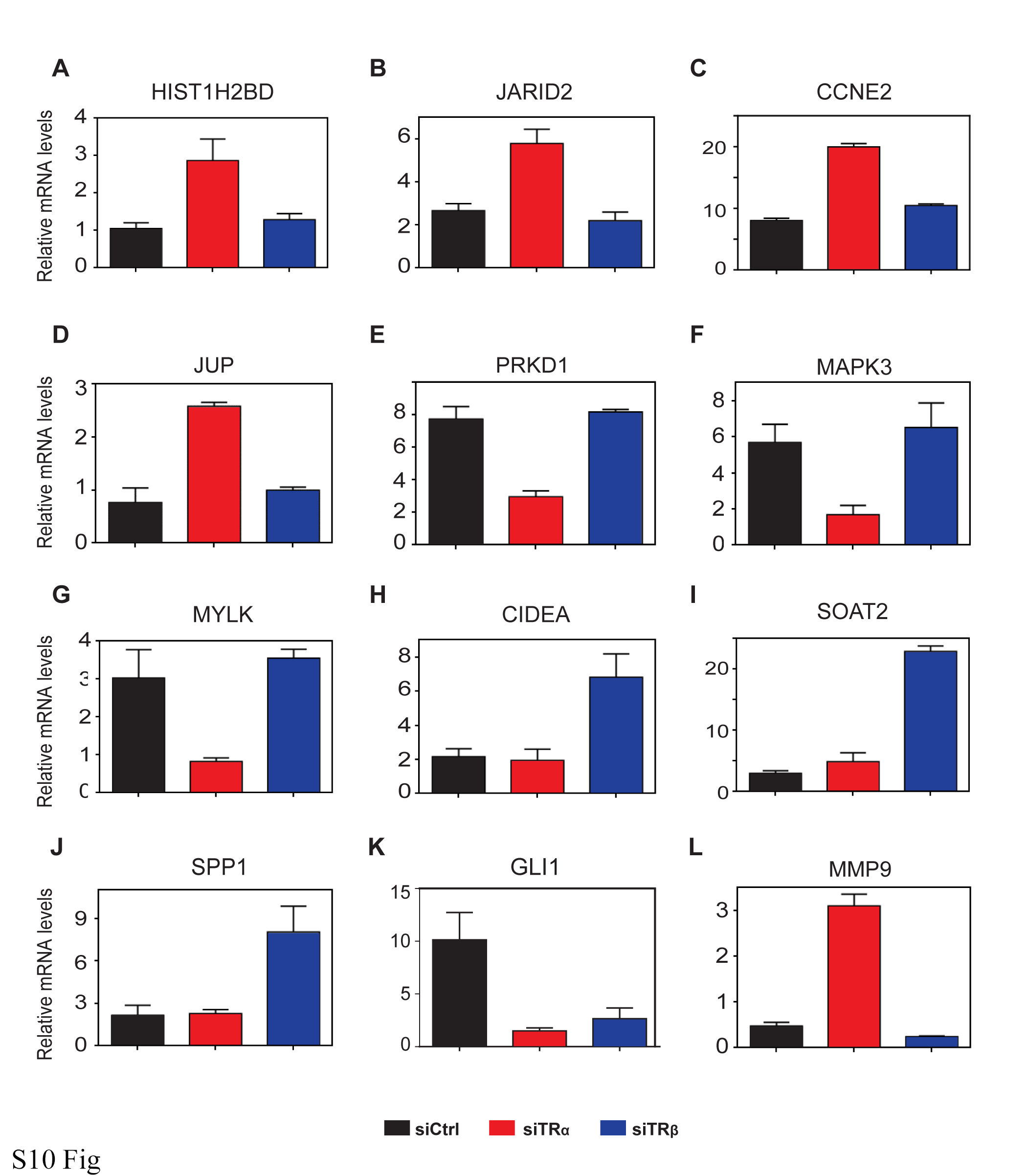

Supplement: S10 Fig — qPCR verification of TRα and/or TRβ target genes as identified by microarray analysis. (TIF) [file pone.0164407.s010.tif]

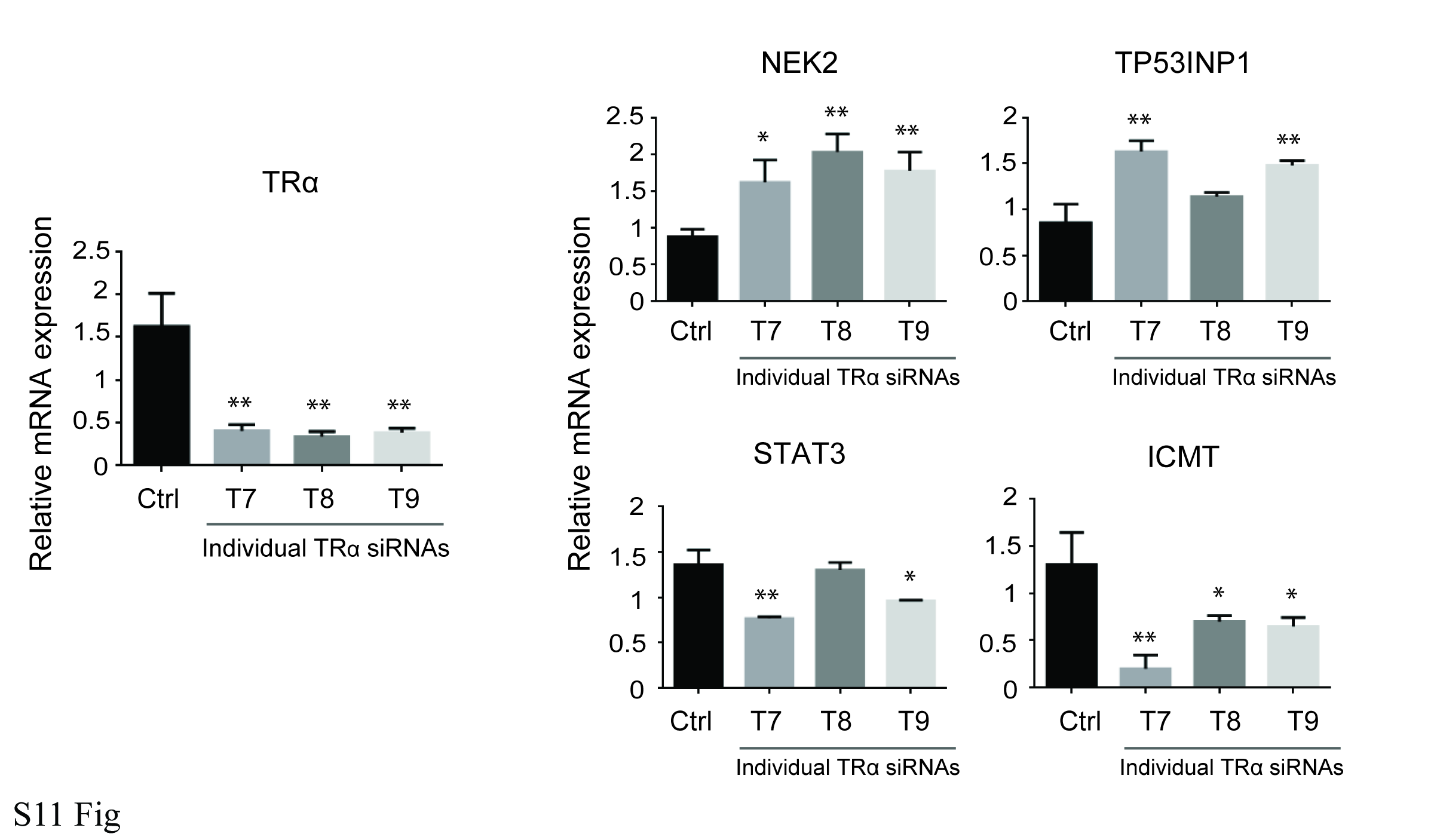

Supplement: S11 Fig — Transcript levels of TRα, NEK2, TP53INP1, STAT3 and ICMT after hADSC were transfected with three different On-TARGET Plus TRα siRNAs at 50 nM final concentration. (TIF) [file pone.0164407.s011.tif]

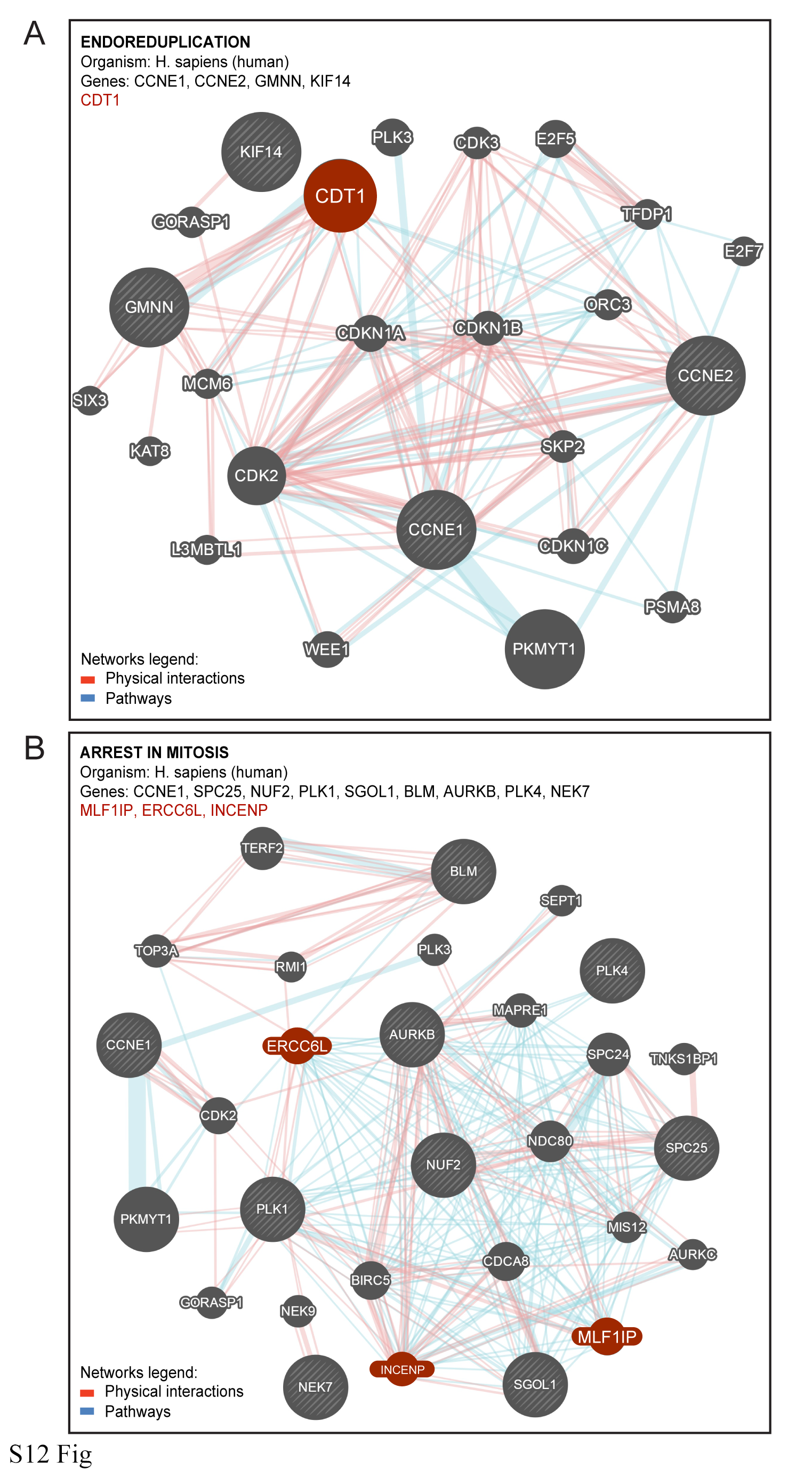

Supplement: S12 Fig — Network of interactions among TRα targets involved in endoreduplication (A) and arrest in mitosis (B), as retrieved by GeneMania. Analysis of network generated with TRα regulated genes involved in these processes (large black circles) revealed genes that are strongly connected to query genes including additional TRα regulated genes (red). (TIF) [file pone.0164407.s012.tif]

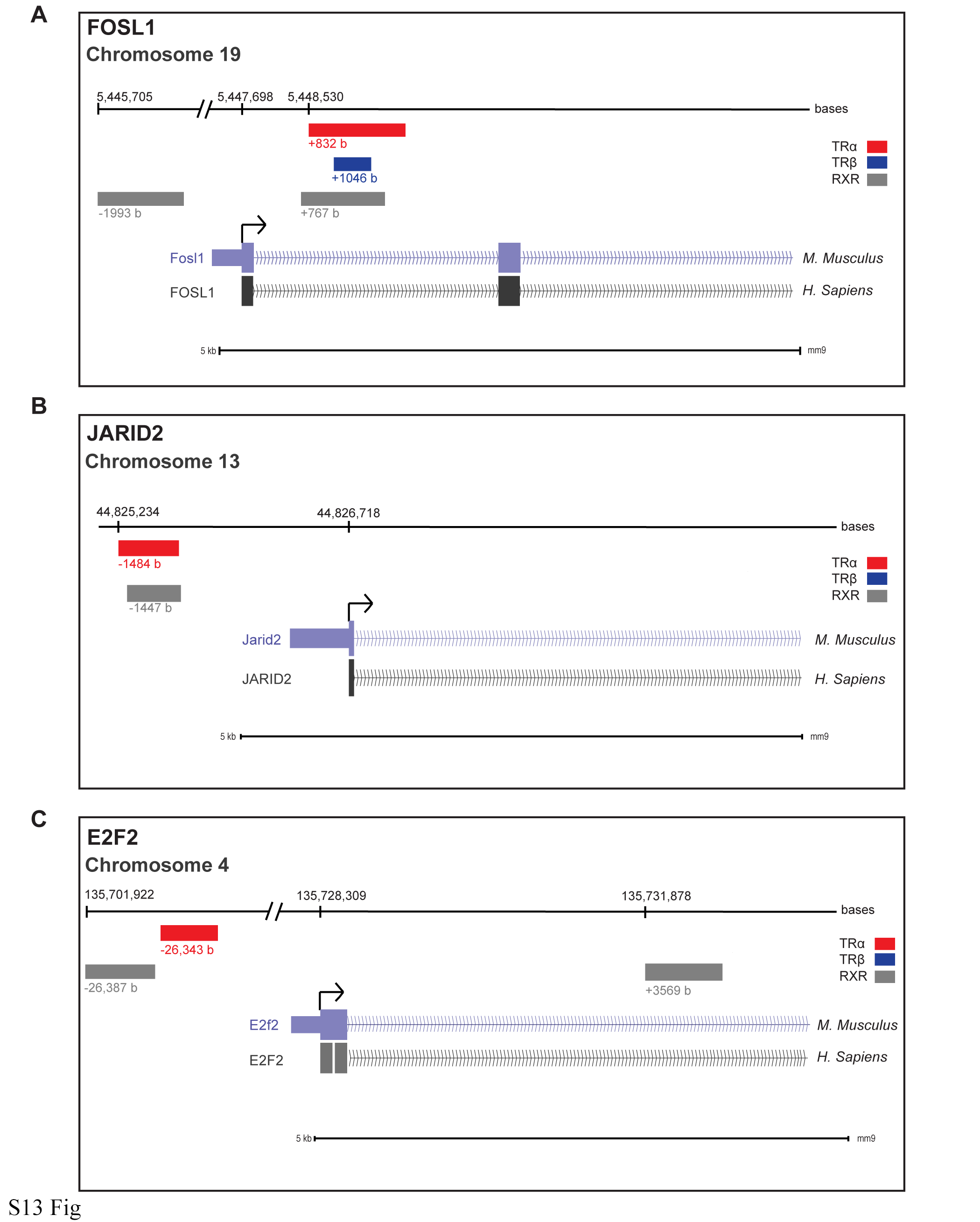

Supplement: S13 Fig — TRα (red bar), TRβ (blue bar) and RXR (grey bar) binding sites located 30K bp upstream to 10K bp downstream of Fosl1, Jarid2 and E2f2, as determined by Chatonnet et al. (17) in mouse neural cells (geodataset series GSE38347). TRα, TRβ and RXR peak bed files were downloaded directly from geodatasets GSM940399, GSM940400, and GSM940401, respectively, and uploaded into the UCSC Genome Browser for viewing and image export. (TIF) [file pone.0164407.s013.tif]

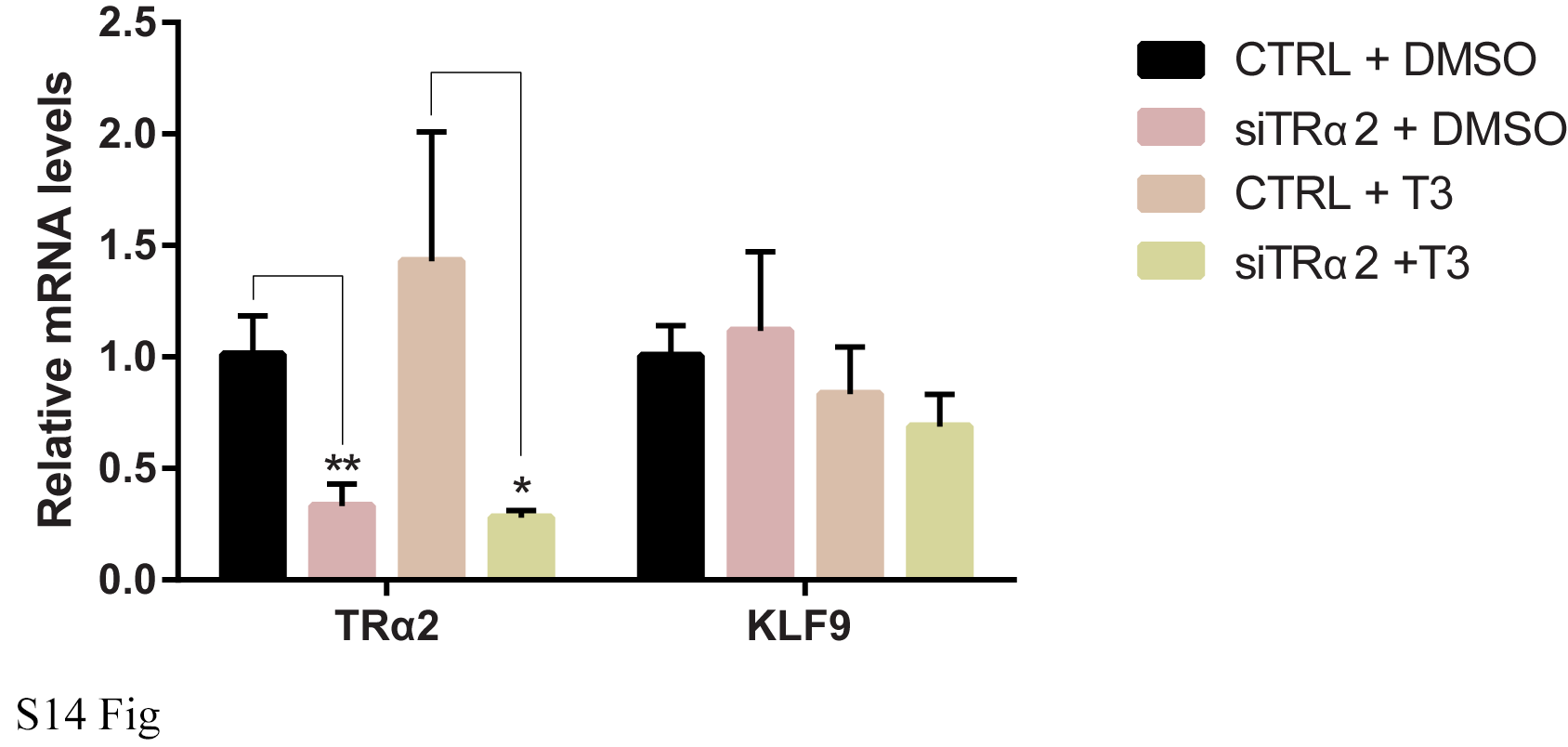

Supplement: S14 Fig — qPCR confirms 50nM siRNA knockdown of TRα2 (A) in hADSCs treated with either DMSO or T3 and shows that KLF9 expression (B) is not significantly changed in either of these conditions. (TIF) [file pone.0164407.s014.tif]
