# Supplementary material for: Ligand Independent and Subtype-Selective Actions of Thyroid Hormone Receptors in Human Adipose Derived Stem Cells
Source: PLoS One. 2016 Oct 12;11(10):e0164407. doi: 10.1371/journal.pone.0164407 (PMC5061422; doi:10.1371/journal.pone.0164407)
Supplement: S1 Table — (DOCX) [file pone.0164407.s015.docx]

**S1 Table.** On-TARGET Plus TR siRNA sequences

| **Target** | **Target Sequences** |
| --- | --- |
| **TRα** (NM_003250; NM_001190918; NM_001190919; NM_199334) | |
|  | GAACCUCCAUCCCACCUAU |
|  | GUAUAUCCCUAGUUACCUG |
|  | GAACUGGGCAAGUCACUCU |
|  | CGGCCAAUGUUCCCUGAAA |
|  |  |
| **TRβ** (NM_001128177; NM_000461; NM_001252634; NM_001128176) | |
|  | UGGAAGUGUUCGAGGAUUA |
|  | GAGAAGAAAUGUAAAGGGU |
|  | GGACAAGCACCAAUAGUCA |
|  | CGAAAUCAGUGCCAGGAAU |
|  |  |
| **TRα1**(NM_199334) |  |
|  | GGAGAAGACAAAUGAAGAA |
|  | GGGAGAAGACAAAUGAAGA |
|  | GAGAAGACAAAUGAAGAAA |
|  | GGAGGAUUGAGAAGGGACA |
|  |  |
| **TRα2** (NM_003250; NM_001190918; NM_001190919) | |
|  | CCAUAAAAGGUGUGUUGAAUU |
|  | CAUAAAAGGUGUGUUGAAUU |
|  | AAGGUGUGUUGAAUUGAAA |
|  | CGUAAGACCACCUUCCCUU |
|  |  |
