# Supplementary material for: Ligand Independent and Subtype-Selective Actions of Thyroid Hormone Receptors in Human Adipose Derived Stem Cells
Source: PLoS One. 2016 Oct 12;11(10):e0164407. doi: 10.1371/journal.pone.0164407 (PMC5061422; doi:10.1371/journal.pone.0164407)
Supplement: S2 Table — (DOCX) [file pone.0164407.s016.docx]

**S2 Table.** Transcription Factors and associated partners were identified among the significantly affected genes through comparison to AnimalTFDB 2.0.

| **Transcription Factors (TF)** | | | **Transcription Cofactors (TCoF)** | |
| --- | --- | --- | --- | --- |
| Symbol | Accession | TF Family | Symbol | Accession |
| KLF15 | NM_014079.2 | zf-C2H2 | AEBP1 | NM_001129.3 |
| KLF9 | NM_001206.2 | zf-C2H2 | BLM | NM_000057.2 |
| ZBTB16 | NM_006006.4 | ZBTB | BTG1 | NM_001731.1 |
| TSC22D3 | NM_001015881.1 | TSC22 | CCNA2 | NM_001237.2 |
| FOS | NM_005252.2 | TF_bZIP | CENPF | NM_016343.3 |
| JUN | NM_002228 | TF_bZIP | CHAF1B | NM_005441.2 |
| NFIL3 | NM_005384.2 | TF_bZIP | CREG1 | NM_003851.2 |
| STAT5A | NM_003152.2 | STAT | PTTG1 | NM_004219.2 |
| NR2F1 | NM_005654.4 | RXR-like | TLE2 | NM_003260.3 |
| MKX | NM_173576.1 | Others | BCL3 | NM_005178.2 |
| LASS6 | NM_203463.1 | Homeobox | WWC1 | NM_015238.1 |
| PITX1 | NM_002653.3 | Homeobox | PPARGC1A | NM_013261.3 |
| PKNOX2 | NM_022062.2 | Homeobox | PIR | NM_001018109.1 |
| SOX13 | NM_005686.2 | HMG | GMNN | NM_015895.3 |
| FOXM1 | NM_202003.1 | Fork head |  |  |
| FOXO1 | NM_002015.3 | Fork head |  |  |
| FOXS1 | NM_004118.3 | Fork head | **Chromatin Remodeling Factors (CRF)** | |
| E2F2 | NM_004091.2 | E2F |  |  |
| E2F7 | NM_203394.2 | E2F | Symbol | Accession |
| CEBPD | NM_005195.3 | C/EBP |  |  |
| MXD3 | NM_031300.2 | bHLH | DNMT1 | NM_001379.1 |
| FUBP1 | NM_003902.3 | Others | EZH2 | NM_152998.1 |
| OSR2 | XM_001126824.1 | zf-C2H2 | SUV39H1 | NM_003173.2 |
| EPAS1 | NM_001430.3 | Others | CBX5 | NM_012117.1 |
| GATAD2A | NM_017660.2 | Others | TBL1X | NM_005647.2 |
| EBF1 | NM_024007.2 | COE |  |  |
| HIC1 | NM_001098202.1 | ZBTB |  |  |
| PRRX2 | NM_016307.3 | Homeobox |  |  |
| TWIST1 | NM_000474.3 | bHLH |  |  |
| GLI2 | NM_005270.3 | zf-C2H2 |  |  |
| MXD4 | NM_006454.2 | bHLH |  |  |
| ZNF281 | NM_012482.3 | zf-C2H2 |  |  |
|  |  |  |  |  |
