# Supplementary material for: Ligand Independent and Subtype-Selective Actions of Thyroid Hormone Receptors in Human Adipose Derived Stem Cells
Source: PLoS One. 2016 Oct 12;11(10):e0164407. doi: 10.1371/journal.pone.0164407 (PMC5061422; doi:10.1371/journal.pone.0164407)
Supplement: S3 Table — In the Nuclear receptors and coregulators PCR array, fold expression differences between hADSC and differentiated cells were analyzed through the SA Biosciences Web page. All experiments were run in triplicates. (DOCX) [file pone.0164407.s017.docx]

**S3 Table.** Change in mRNA expression of NRs after hADSC differentiation.In the Nuclear receptors and coregulators PCR array, fold expression differences between hADSC and differentiated cells were analyzed through the SA Biosciences Web page. All experiments were run in triplicates.

| **NR** | **Adipogenesis (FC)** | **Chondrogenesis (FC)** | **Osteogenesis (FC)** |
| --- | --- | --- | --- |
| RXRA (NR2B1) | 4.0454 | 4.132 | 6.2745 |
| COUP-TFI (NR2F1) | 4.0217 | 3.5743 | 8.8581 |
| THRB (NR1A2) | 6.2454 | 17.3797 | 13.1046 |
| RARG (NR1B3) | -4.6178 | -3.1037 | -2.2110 |
| RARB (NR1B2) | 6.1254 | 3.9614 | -1.0009 |
| RORA (NR1F1) | 2.3179 | 4.1583 | 1.4426 |
| PPARG (NR1C3) | 29.6056 | 1.7996 | 4.2291 |
| GR (NR3C1) | -1.4781 | -2.5312 | -3.9101 |
| AR (NR3C4) | 2.8487 | -1.6546 | 1.5013 |
| ESRRA (NR3B1) | 2.7138 | -1.4639 | -1.3824 |
| LXR (NR1H3) | 15.0929 | -1.5287 | -2.0511 |
| PPARA (NR1C1) | 2.5032 | 1.5996 | 1.6249 |
| THRA (NR1A1) | 2.0803 | 1.0590 | -1.6298 |
| VDR (NR1I1) | -3.1431 | 1.8079 | -1.4369 |
| ESR1 (NR3A1) | 1.9552 | 5.4083 | -1.3368 |
| PPARD (NR1C2) | -1.9395 | -2.4002 | -1.4612 |
| COUP-TFII (NR2F2) | 1.4372 | -2.2786 | 1.1711 |
| MR (NR3C2) | -1.7504 | 2.5814 | 1.3846 |
| EAR1b (NR1D2) | -1.4312 | -1.3698 | -2.1455 |
|  |  |  |  |

***RED***= significant increase in expression levels, FC

***BLUE***= significant decrease in expression levels, FC
