# Supplementary material for: Ligand Independent and Subtype-Selective Actions of Thyroid Hormone Receptors in Human Adipose Derived Stem Cells
Source: PLoS One. 2016 Oct 12;11(10):e0164407. doi: 10.1371/journal.pone.0164407 (PMC5061422; doi:10.1371/journal.pone.0164407)
Supplement: S4 Table — Microarray data are deposited in the Gene Expression Omnibus; accession number GSE75433. (DOCX) [file pone.0164407.s018.docx]

**S4 Table.** Microarray analysis of gene regulation in adipocytes after T3 treatment. Microarray data are deposited in the Gene Expression Omnibus; accession number GSE75433

| **SYMBOL** | **Accession No.** |
| --- | --- |
| **ADIPO** | |
| RUNX1T1 | NM_023072.1 |
| IRX6 | NM_001972.2 |
| ZSWIM4 | NM_144499.1 |
| ELANE | NM_007280.1 |
| GNAT1 | NM_014618.2 |
| OIP5 | NM_007188.2 |
| DBC1 | NM_003512.3 |
| ABCB8 | NM_017738.2 |
| HIST1H2AC | NM_198573.2 |
| CNTLN | XM_935063.1 |
| ENHO | NM_182678.1 |
| RGPD2 | NM_144661.2 |
| UBE2E3 | NM_152498.2 |
| C10orf82 | NM_012324.3 |
| WDR65 | NM_001066.2 |
| MAPK8IP2 | NM_015670.4 |
| TNFRSF1B | XM_945836.1 |
| SENP3 | NM_201564.1 |
| TCP10 | NM_001099784.1 |
| SYCE1 | XM_934475.1 |
| FBXL19 | NM_000208.2 |
| ANAPC11 | NM_020672.1 |
| INSR | NM_001040454.1 |
| S100A14 | NM_002391.3 |
| SLC26A6 | NM_032588.2 |
| MDK | NM_205834.2 |
| TRIM63 | NM_016531.4 |
| LSR | NM_013386.3 |
| KLF3 | NM_145759.2 |
| SLC25A24 | XM_928682.1 |
| TRAF5 | NM_031914.2 |
| KIAA1109 | NM_005892.3 |
| SYT16 | NM_198219.1 |
| FMNL1 | NM_014350.2 |
| ING1 | NM_004665.2 |
| TNFAIP8 | NM_025230.3 |
| VNN2 | NM_001099408.1 |
| WDR23 | NM_144601.2 |
| EIF4E1B | NM_173479.3 |
| CMTM3 | NM_181443.1 |
| WDR88 | NM_004852.2 |
| BTBD3 | XM_926657.1 |
| ONECUT2 | NM_004783.2 |
| CT47B1 | NM_020377.2 |
| TAOK2 | NM_000166.4 |
| CYSLTR2 | XM_929404.1 |
| GJB1 | NM_000771.2 |
| CLGN | NM_004418.2 |
| CYP2C9 | NM_000449.3 |
| DUSP2 | NM_003627.4 |
| RFX5 | NM_203440.2 |
| SLC43A1 | NM_005225.1 |
| C10orf4 | NM_016169.2 |
| E2F1 | NM_001888.2 |
| SUFU | NM_033009.1 |
| CRYM | NM_001079514.1 |
| PCBP4 | NM_001025072.2 |
| UBN1 | XM_930881.1 |
| C3orf17 | NM_031914.2 |
| ANKLE2 | NM_012324.3 |

*RED* = UPregulated*BLUE*= DOWNregulated
