# Supplementary material for: Ligand Independent and Subtype-Selective Actions of Thyroid Hormone Receptors in Human Adipose Derived Stem Cells
Source: PLoS One. 2016 Oct 12;11(10):e0164407. doi: 10.1371/journal.pone.0164407 (PMC5061422; doi:10.1371/journal.pone.0164407)
Supplement: S5 Table — Microarray data are deposited in the Gene Expression Omnibus; accession number GSE75433. (DOCX) [file pone.0164407.s019.docx]

**S5 Table.** Microarray analysis of gene regulation in chondrocytes after T3 treatment. Microarray data are deposited in the Gene Expression Omnibus; accession number GSE75433

| **SYMBOL** | **Accession No.** |
| --- | --- |
| **CHONDRO** | |
| DBP | NM_001352.2 |
| AC092718.3 | NM_052892.3 |
| PKD1L2 | NM_001076780.1 |
| C1QTNF1 | NM_198594.1 |
| PDE7B | NM_018945.3 |
| MOBKL2B | NM_024761.3 |
| ELANE | NM_001972.2 |
| NID2 | NM_007361.3 |
| C20orf82 | NM_080826.1 |
| DBC1 | NM_014618.2 |
| IGF1 | NM_000618.2 |
| CMTM8 | NM_178868.3 |
| GABARAPL2 | NM_007285.6 |
| HEG1 | NM_020733.1 |
| MAN1C1 | NM_020379.2 |
| PIM1 | NM_002648.2 |
| NKD2 | NM_033120.2 |
| RASD1 | NM_016084.3 |
| ZNF90 | NM_031218.2 |
| PRODH | NM_016335.2 |
| DCXR | NM_016286.2 |
| LMOD1 | NM_012134.2 |
| PDCL3 | NM_024065.3 |
| MRGPRF | NM_145015.2 |
| CLIC3 | NM_004669.2 |
| FSTL3 | NM_005860.2 |
| KRT7 | NM_005556.3 |
| SYTL2 | NM_206928.1 |
| HSD17B6 | NM_003725.2 |
| CNN1 | NM_001299.4 |
| FILIP1L | NM_182909.2 |
| ST6GALNAC5 | NM_030965.1 |
| RCAN1 | NM_203417.1 |
| MAP3K8 | NM_005204.2 |
| MYH11 | NM_002474.2 |
| DACT1 | NM_016651.5 |
| AC087521.3 | XM_941665.2 |

*RED* = UPregulated*BLUE*= DOWNregulated
