# Supplementary material for: Ligand Independent and Subtype-Selective Actions of Thyroid Hormone Receptors in Human Adipose Derived Stem Cells
Source: PLoS One. 2016 Oct 12;11(10):e0164407. doi: 10.1371/journal.pone.0164407 (PMC5061422; doi:10.1371/journal.pone.0164407)
Supplement: S6 Table — Microarray data are deposited in the Gene Expression Omnibus; accession number GSE75433. (DOCX) [file pone.0164407.s020.docx]

**S6 Table.** Microarray analysis of gene regulation in osteocytes after T3 treatment. Microarray data are deposited in the Gene Expression Omnibus; accession number GSE75433

| **SYMBOL** | **Accession No.** |
| --- | --- |
| **OSTEO** | |
| TNFSF10 | NM_003810.2 |
| DBC1 | NM_014618.2 |
| AC092718.3 | NM_052892.3 |
| DBP | NM_001352.2 |
| ELANE | NM_001972.2 |
| IGF1 | NM_000618.2 |
| PDE7B | NM_018945.3 |
| PKD1L2 | NM_001076780.1 |
| MXRA5 | NM_015419.2 |
| MOBKL2B | NM_024761.3 |
| MAFB | NM_005461.3 |
| CCL7 | NM_006273.2 |
| TP53I11 | NM_006034.2 |
| MAN1C1 | NM_020379.2 |
| CILP | NM_003613.2 |
| FLYWCH2 | NM_138439.1 |
| RASSF4 | NM_032023.3 |
| ALDH1A3 | NM_000693.1 |
| CMTM8 | NM_178868.3 |
| AC063977.1 | NR_002804.1 |
| PPARGC1A | NM_013261.3 |
| FAM43B | NM_207334.1 |
| ANGPTL2 | NM_012098.2 |
| PTPN22 | NM_015967.3 |
| APOE | NM_000041.2 |
| FUT8 | NM_178156.1 |
| ZNF436 | NM_030634.2 |
| LBP | NM_004139.2 |
| STEAP4 | NM_024636.2 |
| CPA4 | NM_016352.2 |
| ZSWIM4 | NM_023072.1 |
| CPB1 | NM_001871.2 |
| APCDD1 | NM_153000.3 |
| CACHD1 | XM_933387.1 |
| PLA2G5 | NM_000929.2 |
| TIGD2 | NM_145715.2 |
| ADA | NM_000022.2 |
| C11orf17 | NM_182901.2 |
| ALOX5AP | NM_001629.2 |
| RHOJ | NM_020663.3 |
| C1orf87 | NM_152377.1 |
| PLK2 | NM_006622.2 |
| SSH2 | NM_033389.2 |
| ST3GAL5 | NM_001042437.1 |
| STARD5 | NM_181900.2 |
| MUC6 | NM_005961.2 |
| C20orf82 | NM_080826.1 |
| CH25H | NM_003956.3 |
| RNF150 | NM_020724.1 |
| DNASE1L3 | NM_004944.2 |
| PIM1 | NM_002648.2 |
| NPTX1 | NM_002522.2 |
| LDB2 | NM_001290.2 |
| RASD1 | NM_016084.3 |
| COL15A1 | NM_001855.3 |
| CRABP2 | NM_001878.2 |
| ZBTB20 | NM_015642.3 |
| C17orf58 | NM_181656.3 |
| C1QTNF1 | NM_198594.1 |
| GNA13 | NM_006572.3 |
| VPS26A | NM_004896.2 |
| PTPRO | NM_030667.1 |
| RAPH1 | NM_213589.1 |
| TXNL1 | NM_004786.1 |
| PPM1K | NM_152542.2 |
| ATP2B1 | NM_001682.2 |
| PKD2 | NM_000297.2 |
| SORBS1 | NM_001034954.1 |
| UGP2 | NM_006759.3 |
| C8orf85 | NM_001025357.1 |
| EXOSC10 | NM_002685.2 |
| ADAMTS1 | NM_006988.3 |
| FZD6 | NM_003506.2 |
| DACT1 | NM_016651.4 |
| PDE4B | NM_002600.3 |
| A2M | NM_000014.4 |
| MAP3K8 | NM_005204.2 |
| PPP1R14A | NM_033256.1 |
| UBA6 | NM_018227.5 |
| CAST | NM_001042445.1 |
| CT47B1 | XM_926657.1 |
| SEMA3C | NM_006379.2 |
| RRM2B | NM_015713.3 |
| PERP | NM_022121.2 |
| LAMA4 | NM_002290.2 |
| SLC40A1 | NM_014585.4 |
| HHIP | NM_022475.1 |
| C12orf23 | NM_152261.1 |
| HERC4 | NM_015601.2 |
| HSPA13 | NM_006948.4 |
| C13orf37 | NM_001071775.1 |
| FBXO38 | NM_030793.3 |
| SPOCD1 | NM_144569.4 |
| ADAM10 | NM_001110.2 |
| MYH11 | NM_002474.2 |
| CHN2 | NM_004067.2 |
| ILK | NM_001014794.1 |
| CCDC132 | NM_017667.2 |
| DKK1 | NM_012242.2 |
| TSC22D1 | NM_183422.1 |
| SSTR1 | NM_001049.2 |
| NDUFAF4 | NM_014165.1 |
| FGF7 | NM_002009.2 |
| ATF4 | NM_182810.1 |
| ZFP36L1 | NM_004926.2 |
| HECA | NM_016217.2 |
| SNX25 | NM_031953.2 |
| FSTL3 | NM_005860.2 |
| B3GALT2 | NM_003783.2 |
| NRCAM | NM_005010.3 |
| FSIP1 | NM_152597.4 |
| ID2 | NM_002166.4 |
| TFPI2 | NM_006528.2 |
| MYPN | NM_032578.2 |
| RHOBTB1 | NM_198225.1 |
| SLC39A8 | NM_022154.5 |
| CD302 | NM_014880.3 |
| HMGCS1 | NM_002130.6 |
| FN1 | NM_054034.2 |
| CCBP2 | NM_001296.3 |
| HNRNPH2 | NM_001032393.1 |
| DNAJB4 | NM_007034.3 |
| LIFR | NM_002310.3 |
| MYADM | NM_138373.3 |
| MBNL2 | NM_144778.2 |
| COL10A1 | NM_000493.3 |
| ACAN | XM_938439.1 |
| HSPA2 | NM_021979.2 |
| FAM167A | NM_053279.1 |
| C5orf46 | NM_206966.2 |
| GREM1 | NM_013372.5 |
| CDKN1C | NM_000076.1 |
| TELO2 | NM_016111.2 |
| PTX3 | NM_002852.2 |
| POSTN | NM_006475.1 |
| CLIC3 | NM_004669.2 |
| AC087521.3 | XM_941665.2 |

*RED* = UPregulated*BLUE*= DOWNregulated
