# Supplementary material for: Ligand Independent and Subtype-Selective Actions of Thyroid Hormone Receptors in Human Adipose Derived Stem Cells
Source: PLoS One. 2016 Oct 12;11(10):e0164407. doi: 10.1371/journal.pone.0164407 (PMC5061422; doi:10.1371/journal.pone.0164407)
Supplement: S7 Table — (DOCX) [file pone.0164407.s021.docx]

**S7 Table.** Differentially expressed genes in the TRα and TRβ-mediated processes obtained from GeneCodis using SlimProcess database (Table 2).

| Annotations | Gene Symbol |
| --- | --- |
| TRα |  |
| GO:0007267 cell-cell signaling (BP) | CXCL6,GJB2,WNT7B,BMP2,CXCL10,CCL7,CXCL5,NAMPT,WNT2,SSTR1,ISG15,STC2,GPR56,TFAP2C,KLF10,STC1,NTF3,EFNB2,  CCL8,PTGIR,FGF18 |
| GO:0007165 signal transduction (BP ) | CXCL6,TNFRSF10D,NGF,CLIC3,ARHGAP22,DEPDC1,STAC,  CHRNA5,CXCL10,CCL7,CXCL5,STAT3,MX1,RASD1,EPHB1,  NAMPT,ADRA2A,IRAK2,GUCY1A2,EDNRA,IL1RAPL1,CSF1R,IL8,ITPKA,CYTL1,RASL12,STK24,PDE4B,RCAN1,HMOX1,ANGPTL4,  PRKD1,PRKCZ,NTF3,ARHGAP28,STMN1,GRB14,CCL8,  TNFRSF10A,PDE1C,PDE1A,RASSF7,FGF18 |
| GO:0007067 mitosis (BP) | NEK2,HAUS8,CDCA3,UBE2I,CDC6,HELLS,ANLN,NUF2,PLK1,  CDC25A,HGF,PBK,MASTL |
| GO:0051301 cell division (BP) | CCNE2,NEK2,HAUS8,SGOL1,CCNE1,KIFC1,CDCA3,UBE2I,CDC6,SPC25,HELLS,NUF2,CDC25A,ERCC6L,MASTL,PARD6G |
| GO:0008283 cell proliferation (BP) | TACSTD2,MCM7,STAT3,AURKB,IL1A,TACC3,EDNRA,CSF1R,  KLF10,GRPR,PRKD1,PLK1,CDC25A,DAB2,CDK5R1,PIM1 |
| GO:0007059 chromosome segregation (BP) | NEK2,SGOL1,UBE2I,SPC25,NUF2,HJURP,INCENP |
| GO:0055085 transmembrane transport (BP) | SLC38A1,DST,SLC29A1,SLC25A29,SLC25A13,CACNA1H,  SLC22A12,HMOX2,SLC6A6,SLC24A6,SLC16A6,TMCO3,SLC11A2,SLC1A5,SLC6A9,HMOX1,SLC7A11,SLC8A3,SLC43A3,SLC25A24,  SLC5A3,SLC16A9,SLC45A3 |
| GO:0006950 response to stress (BP) | NDRG4,MAPK13,HSPB6,TRIB3,HSPB7,TP53INP1,TACC3,HSPA6,  MAFF,PYCR1 |
| GO:0040007 growth (BP) | BMP2,SEPP1,INHBE,BMP4,GDF10 |
| GO:0007155 cell adhesion (BP) | LEF1,HAS1,F8,DST,ITGA3,ITGA2,HAPLN1,ITGA5,GPR56,  COL22A1,CADM4,CXADR,PRKX,OMD,EFNB2,PCDH18,COL11A1,EMR2,ITGA11,OPCML,THBS3 |
| GO:0034641 cellular nitrogen compound metabolic process (BP) | ARG2,SLC38A1,ASS1,KYNU,SLC6A6,SLC1A5,TDO2,SLC7A11,  PYCR1,MCCC2,GLS |
| GO:0007049 cell cycle (BP) | DIXDC1,FANCI,MAPK3,MAPK13,HAUS8,KIFC1,CDCA3,AURKB,  UBE2I,HELLS,ANLN,HJURP,GSG2,LIN9,PIM1,MCM2,PARD6G |
| GO:0030198 ECM organization (BP) | ECM2,MMP9,CCDC80,APLP2,KAZALD1,COL11A1 |
| GO:0030154 cell differentiation (BP) | SLIT3,TAF7L,NDRG4,ARHGAP22,PAQR5,NTNG1,DUSP6,  KAZALD1,HES4,MEG3,ANGPTL4,PLD6,BEX1,STMN1,INA,EFNB2,MGP |
| GO:0006464 protein modification process (BP) | HERC5,ST3GAL6,UBE2I,ICMT,ST6GAL1,LOX,FBXO2 |
| GO:0009790 embryo development (BP) | BMP2,FOXQ1,APLP2,FZD4,CDK5R1,PSEN2 |
|  |  |
| TRβ |  |
| GO:0007267 cell-cell signaling (BP) | IL1B,GJB2,BMP2,CXCL10,CCL7,CXCL5,ISG15,GPR56,SFRP2,KLF10,STC1,EFNB2 |
| GO:0007155 cell adhesion (BP) | HAS1,DPT,ITGA3,SVEP1,ITGA2,HAPLN1,SPP1,GPR56,COL22A1,  PTPRU,AJAP1,OMD,CXCL12,EFNB2,EMR2,OPCML |
| GO:0007165 signal transduction (BP ) | IL1B,TNFSF13B,ARHGAP22,CXCL10,CCL7,CXCL5,MX1,EPHB1,  DIRAS3,IL1RAPL1,HBEGF,CSF1R,IL8,SFRP2,ANGPTL4,SH3GL3,  RRAD,ARHGAP28,CXCL12,GRB14,ARRDC3,PLAU |
| GO:0008283 cell proliferation (BP) | PRG4,TNFSF13B,CLK1,TACSTD2,ERCC1,IL1A,CSF1R,KLF10,GRPR,CDC25A |
| GO:0030154 cell differentiation (BP) | ARHGAP22,PAQR5,SEMA7A,FCRLA,SLC26A8,GAP43,SFRP2,PTPRU,MEG3,ANGPTL4,BEX1,INA,EFNB2 |
| GO:0009790 embryo development (BP) | BMP2,FOXQ1,TPM1,SFRP2,PSEN2,PSEN1 |
| GO:0055085 transmembrane transport (BP) | HK2,ADCY1,SLC38A1,SLC29A1,SLC22A12,SLC26A8,SLC4A4,  SLC16A6,TPR,SLC43A3,SLC5A3 |
