# Supplementary material for: Ligand Independent and Subtype-Selective Actions of Thyroid Hormone Receptors in Human Adipose Derived Stem Cells
Source: PLoS One. 2016 Oct 12;11(10):e0164407. doi: 10.1371/journal.pone.0164407 (PMC5061422; doi:10.1371/journal.pone.0164407)
Supplement: S8 Table — (DOCX) [file pone.0164407.s022.docx]

**S8 Table.** List of the differentially expressed genes after TRα or TRβ knockdown in the enriched cell cycle-related functional themes (IPA) in hADSC (Table 3).

| Function annotation | Gene symbol |
| --- | --- |
| TRα |  |
| Cell cycle progression | ANLN, AURKB, BLM, BMP2, BMP4, BTG2,CCNE1, CCNE2, CD24, CDC25A, CDC6, CDK5R1, COPZ2, CSF1R, CSF3, CXCL10, CXCL8, DIXDC1,DNMT3B, E2F2, FOS, GLI1, GMNN, HAS1, HAUS8, HELLS, HERC5, HGF, HMOX1, IL1A, INCENP, IRF7, ITGA2, JARID2, KIFC1, LEF1, LIN9, MAFB, MAPK3, MASTL, MCM2, MCM7, mir-21, mir-25, MYH14, NEK2, NEK7, NGF, NTF3, NUF2, ORC6, PIM1, PLK1, PLK4, POLD1, PRKCZ, PSEN2, PTX3, SGOL1, SPC25, STAT3, STMN1, TACC3, TP53INP1, UBE2I,VASH2, ZAK |
| S phase | BLM, CCNE1, CDC25A, CDC6,CSF1R, E2F2, FEN1, FOS, H19,HGF, HMOX1, ITGA2, MAPK3, MCM10, MCM7, mir-21, PLK1, PMEPA1, PPAP2C, PSEN2, SSTR1, STAT3 |
| Entry into S phase | CCNE1, CDC25A, CDC6, E2F2, FOS, H19, HMOX1, ITGA2, MCM10,MCM7, PPAP2C, SSTR1, STAT3 |
| Re-entry into cell cycle progression of tumor cell lines | E2F2, NGF, PLK1 |
| M phase | ANLN, AURKB, CCNE1, CDC6, CDT1, CYP26B1, INCENP,KIF14, KIFC1, MASTL, MCM7, mir-10, MYH14, NEK2, NEK7, NUF2, PIM1, PLK1, TM4SF1 |
| Arrest in M phase | CCNE1, CDT1, CYP26B1, KIFC1, MASTL, NEK7, NUF2, PLK1 |
| Mitosis | ANLN, AURKB, BLM, BMP2, BMP4, CCNE1, CDC25A, CSF1R, CXCL10, E2F2, FOS, GMNN, HAUS8, HELLS, HGF, IL1A, INCENP, JARID2, KIFC1, LEF1,MAPK3, MASTL, MYH14, NEK2, NEK7, NGF, NTF3, NUF2, ORC6, PLK1, PLK4, PRKCZ, PTX3, SGOL1, SPC25, STAT3, STMN1 |
| Arrest in mitosis | AURKB, BLM, CCNE1, NEK7, NUF2, PLK1, PLK4, SGOL1, SPC25 |
| Arrest in interphase | BLM, BMP2, BMP4, BTG2, CCNE1, CDC25A, CDC6, CSF1R, CSF3, E2F2, FEN1, FOS, GMNN, GSG2, HGF, IL1A, ITGA5, KLK3, MAPK3, MASTL, MCM7, mir-21, mir-25, MMP9, NGF, NUAK1, PIM1, PLK1, PPAP2C, PSEN2, SSTR1, ZAK |
| Interphase | BLM, BMP2, BMP4, BTG2, CCNE1,CCNE2, CDC25A, CDC6, CSF1R, CSF3, E2F2, FEN1, FOS, GMNN, GSG2, H19, HGF, HMOX1, IL1A, ITGA2, ITGA5, KLK3, LEF1, MAPK3, MASTL, MCM10, MCM7, mir-21, mir-25, MMP9, NGF, NUAK1, PIM1, PLK1, PMEPA1, PPAP2C, PSEN2, SSTR1, STAT3, TPD52L1, ZAK |
| Arrest in G1 phase | BMP2, BTG2, CCNE1, CDC25A, CDC6, CSF1R, CSF3, E2F2, GMNN, GSG2, IL1A, ITGA5, KLK3, MAPK3, MCM7, mir-25, NGF, NUAK1, PIM1, PSEN2, SSTR1 |
| G1 phase | BMP2, BTG2, CCNE1, CCNE2, CDC25A, CDC6, CSF1R, CSF3, E2F2, GMNN, GSG2, HGF, IL1A, ITGA5, KLK3, LEF1, MAPK3, MCM7, mir-25, NGF, NUAK1, PIM1, PSEN2, SSTR1, STAT3 |
| Cytokinesis | ANLN, AURKB, CDC6, INCENP, KIF14,KIFC1, MASTL, MYH14, NEK2,NEK7, PIM1, PLK1, TM4SF1 |
| Arrest in prometaphase | CCNE1, CDT1, KIFC1, MASTL, NUF2 |
| Senescence of cells | BLM, BMP4, CCNE1, DDB2, DNMT3B,HELLS, HGF, HJURP, IL1A, LIN9, MAPK3, mir-25, NAMPT, PHC2, PLK1, PRKD1, SCMH1, TM4SF1, TSC22D1 |
| Replication of bone marrow cells | AURKB, CCNE1, CCNE2 |
| Formation of mitotic spindle | HAUS8, HELLS, KIFC1, NEK2, NEK7, NUF2, PLK1, STMN1 |
| Premature senescence of fibroblasts | DDB2, DNMT3B, LIN9, PHC2 |
| Endoreduplication | CCNE1, CCNE2, GMNN, KIF14 |
|  |  |
| TRβ |  |
| Cell cycle progression | BLM, BMP2, CDC25A, CSF1R, CXCL10, CXCL12, CXCL8, DDX17, DIRAS3, FOSL1, GLI1, HAS1, HBEGF, HERC5, IL1A, IL1B, IRF7, ITGA2, KRT19,mir-21, MX2, MYH14,NEK7, PDCD1LG2, PSEN1, PSEN2, PTX3, RRAD,SPP1, TBRG1, TMEFF2, TNFSF13B, TPR, TRIM21, ZNF365 |
| S phase | BLM, CDC25A, CSF1R, CXCL12, H19,HBEGF, HMGA1, IL1B, ITGA2, mir-21, PSEN1, PSEN2, TRIM21 |
| Entry into S phase | CDC25A, CXCL12, H19, HBEGF, HMGA1, IL1B, ITGA2, PSEN1, TRIM21 |

***RED*** = significant increase after KD; ***BLUE***= significant decrease after knockdown
